# Recent patent applications in beverages enriched with plant proteins

**DOI:** 10.1038/s41538-021-00112-4

**Published:** 2021-11-01

**Authors:** Clara Takayama Arbach, Izabel Almeida Alves, Mairim Russo Serafini, Rodrigo Stephani, Ítalo Tuler Perrone, Juliana de Carvalho da Costa

**Affiliations:** 1 Nutrition Department, Federal University of Juiz de Fora, Juiz de Fora, Minas Gerais Brazil; 2 Medical School Department, Federal University of Bahia, Salvador, Bahia Brazil; 3 Pharmacy Department, Federal University of Sergipe, São Cristóvão, Sergipe Brazil; 4 Chemistry Department, Federal University of Juiz de Fora, Juiz de Fora, Minas Gerais Brazil; 5 Pharmaceutical Sciences Department, Federal University of Juiz de Fora, Juiz de Fora, Minas Gerais Brazil

**Keywords:** Nutrition, Science, technology and society, Sustainability

## Abstract

Recently, many consumers have been adding plant-based beverages to their diets, due to different reasons. The addition of plant proteins to enrich these products in order to make them more nutritionally balanced has become a trend, mainly because of their lower prices and reduced environmental damage. Thus, the aims of the present patent review are to discuss the potential of, and challenges posed by, plant proteins to the beverage industry, as well as to check market trends, focused on raw materials and beverage types. Based on the results, pea, rapeseed, bean, peanut, chickpea, lentil, hempseed, sunflower seed, and cottonseed were among the most often addressed raw materials. Furthermore, this enrichment process is not limited to create products that mimic dairy, therefore expansion in plant proteins used to enrich carbonated beverages, sports drinks, or even juices is expected to happen. Thus, plant-derived proteins have been promising to high-quality beverage production, as well as to ensure food security, animal welfare, and low environmental impacts.

## Introduction

Nowadays, plant-based beverage consumption has been gaining significant relevance. This segment has been projected to exceed US$26 billion by 2023, in the global market^1^. Environmental concerns and ethical awareness about animal welfare are factors often pointed out as motivation for the consumption of plant-based products, as well as a preference for a healthy lifestyle^2^. In fact, plant matrices tend to present a higher amount of fiber and phytochemicals, when compared to animal matrices. One more reason for the health-promoting effect of a plant-based diet is linked to the lifestyle as a whole, since those who opt for this diet are likely to be more conscious regarding food and exercise habits, and as a result, acquire protective factors against chronic diseases^3^. Moreover, the increasing incidence of individuals allergic to cow milk and intolerant to lactose mostly account for such consumption shift^2^.

A wide range of plant-based products is available in the market, mainly when it comes to milk analogs. In technological terms, milk analogs are aqueous extracts from milled plant material that resembles cow milk^4^. Soy-based beverages were the first of these products to become popular; however, they are currently facing rejection due to their allergenicity and genetic modifications^5–8^. At the same time, other plant sources have emerged as alternatives to soy-based beverages, as among them one finds almond, rice, oat, coconut, sunflower seed, and hempseed^1^. Because these raw materials are different from each other, the nutritional composition, appearance, and taste of final products can present considerable differences^1,6^. Regarding nutrients, for example, protein is a nutrient that tends to be missing in these beverages, and this issue can limit their acceptance by consumers^8^.

Indeed, people have been seeking protein-rich products^9^. According to Mintel, 30% of consumers would buy more milk substitutes if they had an extra protein appeal^10^. Plant source blends have been explored as the way to accomplish the nutritional adequacy and improvement of final products’ technological aspects^11,12^. However, the enrichment of products with proteins has become a major trend for this new beverage in the market, in order to make them even more nutritionally balanced^9^. Such enrichment can be applicable not only to milk replacers, but also to other sorts of beverages, such as fermented beverages, sports drinks, soft drinks, or even juices, in case appropriate technologies are applied to their production process.

Legumes, pulses, seeds, and nuts are often highlighted as great sources of plant protein, mainly oil industry by-products, like press-cakes, which are protein-rich, but yet poorly explored as food^13,14^. Leaves, aquatic plants, seaweed, and microalgae can also be used as raw materials for protein extraction. However, they require processing near the growing area, due to their high moisture and rapid deterioration^15^. When correctly combined, plant proteins are comparable to animal-based proteins, as they can provide all essential amino acids in adequate amounts^16^. It is also known that di and tripeptides can be absorbed by the gut, and, as well as animal proteins, plant proteins can be hydrolyzed in biopeptides which present health-promoting effects. Some of these benefits are^17,18^, for example, anticancer, antimicrobial, antioxidant, anti-inflammatory, antianemic, antithrombotic, antihypertension, antiobesity, antidiabetic, and immunomodulatory activities^19,20^. These biopeptides normally do not present bioactivity when encrypted in the parent protein, so the benefit only displays after the hydrolysis process^21^. Depending on the peptide, they can be obtained by natural digestion process or by food processing, using trypsin, pepsin, alkalase, and biological fermentation to create functional peptide ingredients^22^. Besides that, plant proteins account for costs lower than animal proteins; moreover, their production process is less damaging to the environment^13^. On the other hand, dealing with plant proteins can be challenging, since their functionality-related aspects, such as color and taste, remain an issue. Furthermore, even if the total amount of protein is high, the presence of limiting amino acids and antinutritional factors in plants ends up compromising the quality of the protein^15^.

But as the challenges related to plant proteins use have been overcome and their potential has been perceived, patenting mechanisms now play an important role. The existence of patenting mechanisms is crucial in the current technological world because it allows exclusivity to use an invention for a certain period of time. However, it happens in exchange for the disclosure of detailed information about the topic^23^. Any technical or functional aspect of products and processes can be patented, as long as they meet industrial applicability, novelty, inventiveness, and patentable subject matter criteria^24^. Plant proteins properties modifications, specific temperature, and pH conditions used in processes, isolation, and purifying methods, as well as the final product itself can be protected by patents^25^. More than working as a competitive instrument, patents are valuable sources of technological information^23^; therefore, the aims of the current patent review were to discuss the potential of, and challenges posed by, certain plant proteins to the beverage industry, as well as to check market trends focused on raw materials and beverage types.

## Methodology

**Fig. 1:**
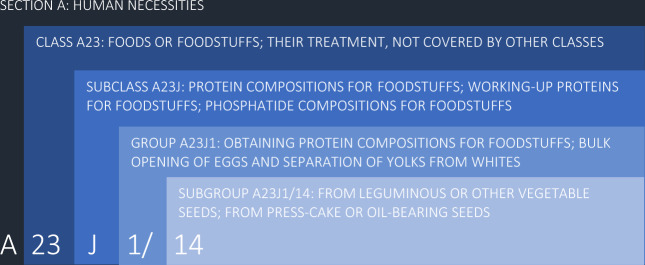
Definition of subgroup A23J1/14 based on the International Patent Classification (IPC). Darker blue categories represent broader fields, whereas lighter blue categories concern more specific fields. Data source: World Intellectual Property Organization (WIPO). Elaborated by the authors.

The search for patents was based on the International Patent Classification (IPC) system and carried out in “Espacenet” since this database gathers the largest number of open access patents. It is better to use a patent classification system rather than the “keyword” strategy alone because it ensures the selection of patents belonging to a specific field and technology. Patents published between January 2015 and June 2020 were herein selected in association with the IPC subgroup A23J1/14, which describes the technologies and applicability of protein compositions for foodstuffs obtained from leguminous, other vegetable seeds, press-cake, or oil-bearing seeds (Fig. 1).

Patents presenting duplicate or triplicated titles applied by the same authors and coming from different origins were excluded from the research as part of the screening. It is important taking into consideration that when duplicated or triplicated patents were excluded, the newest ones were maintained in the sample. Patents that were presented in title terms such as “soy”, “hybrid”, or “variety” were also excluded from the study. Soy was excluded given its growing rejection by consumers, mainly because of its allergenicity and genetic modifications^8^. Likewise, titles with the words “hybrid” and “variety” were also excluded, since they are related to the production of new plants based on genetic engineering, which is not the aim of the current study.

The full reading of the patents was carried out and the following eligibility criteria were established to exclude: (1) patents with an unavailable description or no translation into English or Portuguese; (2) duplicated or triplicated patents by the same authors, from different origins; (3) patents that used soy as preferable raw material; and (4) patents that used animal origin components as preferable raw material.

**Fig. 2:**
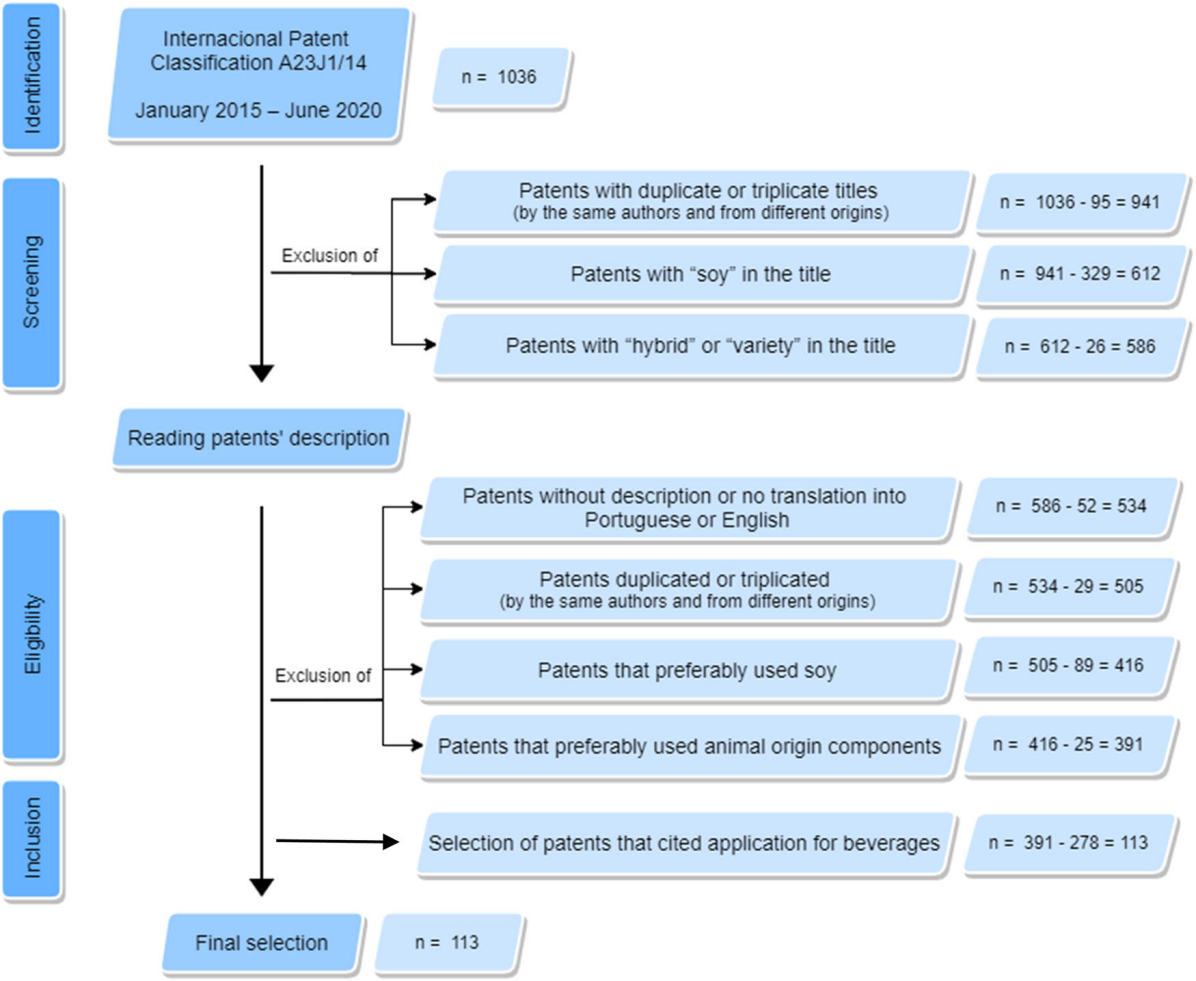
Research methodology flowchart. Patents’ identification followed by screening, and eligibility and inclusion criteria application.

The keyword search was carried out in the descriptions by combining the following terms: “beverage” or “juice” or “drink” or ((“dairy” or “milk” or “yogurt”) and (“alternative” or “substitute” or “analog” or “replacer”)) in order to only select patents related to plant-based beverages. All surveyed patents were somehow related to plant proteins used as ingredient and/or supplement, and/or additives in the beverage industry, or even as a ready-to-consumption product (Fig. 2).

## Results and discussion

**Fig. 3:**
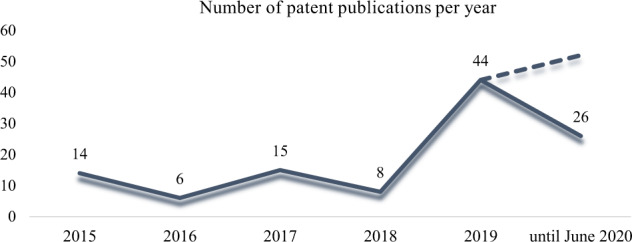
Number of patent publications per year. Solid line refers to the current number of filled patents over time; dashed line means the estimate for 2020 by taking into consideration the increasing number recorded in the previous year and in early 2020.

In total, 1036 patent applications were found from January 2015 to June 2020 in the IPC A23J1/14 subgroup. The final sample counted on 113 patents after screening and eligibility criteria application. By comparing the number of plant-based beverage applications in the last 5 years, it was possible seeing an increase in this number back in 2019: 44 patent applications (Fig. 3). Noteworthy, 26 patents were applied up to June 2019, and this finding is indicative of upward tendency estimated to the number of applications in the following years. Such an estimate can be confirmed by both the current plant-based beverage consumption trend^1^ and populations’ increased interest in protein-rich products^9^.

**Fig. 4:**
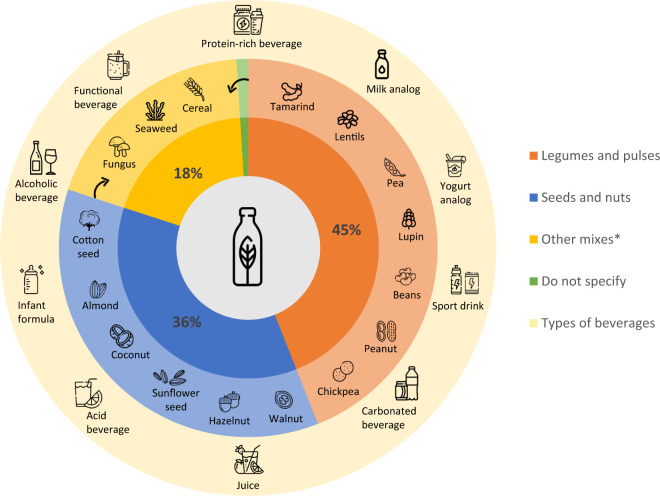
Distribution of raw material groups and beverage types that can be produced. Legumes and pulses examples are highlighted in orange; seeds and nuts, in blue; other mixes, in yellow; do not specify, in green; beverage types are expressed in light yellow. *Other mixes include patents that used “legumes and pulses” and/or “seeds and nuts” and/or other components, such as cereal, seaweed, and fungus.

Patents were divided into three different groups, namely: “seeds and nuts”, “legumes and pulses”, and “other mixes”, since A23J1/14 subgroup is related to leguminous, vegetable seeds, press-cake, or oil-bearing seeds (Fig. 4). Based on the results, most patents are related to legumes and pulses using (45%), followed by seeds and nuts (36%), other mixes (18%), and patents that did not specify their raw materials (1%).

**Fig. 5:**
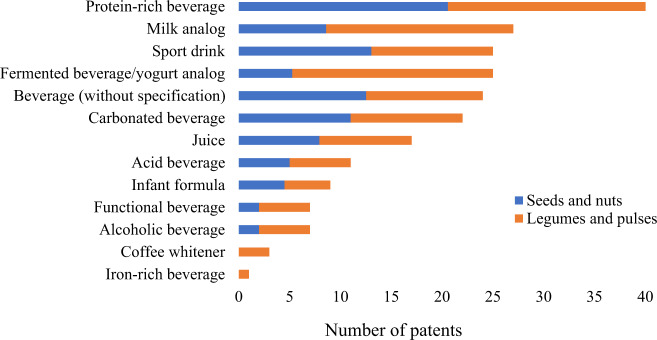
Number of patents based on beverage types and raw material groups. Patents that have opted for seeds and nuts are represented in blue, whereas the number of patents that have used legumes and pulses is represented in orange. *The same patent can mention more than one beverage type as applicable.

Most patents (*n* = 40) mentioned their applicability to protein-rich beverage production. This finding was expected due to the current demand for this category of product^9^ (Fig. 5). Milk analog (*n* = 27) and fermented beverage/yogurt analog (*n* = 25) come right in sequence, and it corroborated consumers’ increased interest in alternatives to dairy products^1^. Sports drink (*n* = 25), carbonated beverage (*n* = 22), juice (*n* = 17), and acid beverage (*n* = 11) were also often addressed in patents, and this finding meets the current need of using plant proteins at different pH ranges without compromising the features of final products.

**Fig. 6:**
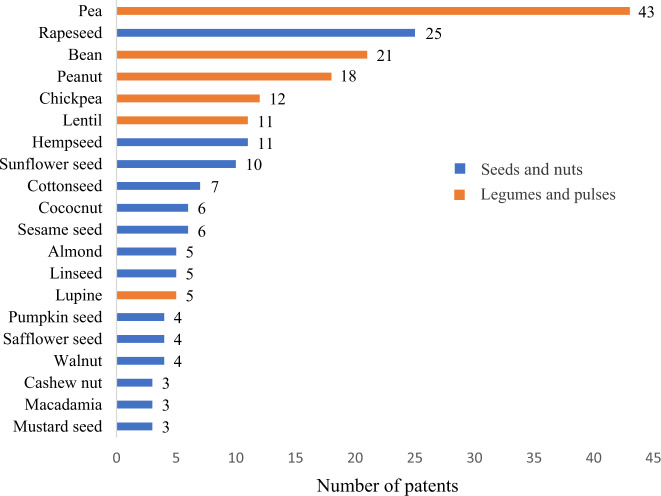
Number of patents by preferable raw materials for use. Seeds and nuts are represented in blue, whereas legumes and pulses are represented in orange. *The same patent can use more than one raw material as preferable; **In total, 13 patents used other raw material types, such as cereal, seaweed, or fungus, besides using legumes, pulses, seeds, or nuts; ***Three (3) patents did not specify the used raw materials; ****Brazil nut, Camelina seed, Castor bean, Chia seed, Cocoa seed, Fenugreek, Ginkgo biloba seed, Pepper seed, Perilla seed, Physalis seed, Pistachio, Primula seed, Samara seed, Sea buckthorn seed, Snackegourd seed, Tamarind, Thistle seed, Tomato seed, Tung seed, Turnip seed, and Wisteria were cited as preferable raw material by one patent. *****Alfalfa, Broccoli seed, Camellia seed, Grape seed, Hazelnut, Moringa oleifera seed, and Locust bean were cited as preferable raw material by two patents.

Figure 6 shows the preferable raw materials for patent applicants. It is important to point out that the sum of recorded results will be higher than the total number of patents selected for the current study if one takes into consideration that some patents used more than one raw material on its applicability. The most preferable raw materials chosen by patents applicants were pea, rapeseed, and bean.

### Seeds and nuts

Seeds are of great economic and biological importance if one takes into account that they have a high oil and protein content, and large starch reserves. Nuts from trees are recommended as part of a healthy diet given their protein, phytochemicals, micronutrients, and unsaturated fat content^14^. Seeds and nuts can be the raw material for oil production, which is often extracted by cold pressing—this process generates a by-product known as “press-cake”; or it can also be extracted with organic solvents—in this case, the residue is called “meal”^26^. The global 2018/2019 oilseed production reached 600.47 million metric tons^27^, hence huge amounts of oil extraction by-products are available. Although press-cakes and meals are often discarded as waste or used as components for feed supplementation, they are good protein, fiber, and bioactive compound sources^26,28^. Moreover, because of the increasing demand of protein products, such waste types are a potential alternative source for protein extraction^5^. However, the oil extraction procedure often decreases protein solubility^29^ and it compromises its use in beverages. Thus, many of the herein-reviewed patents were related to methods applied to increase or preserve the solubility of proteins extracted from cakes and meals. Furthermore, depending on the raw materials, meals and press-cake often possess undesirable amounts of compounds that decrease protein digestibility, such as fibers and antinutritional factors^5^—such a matter is constantly discussed in most patents as well. Moreover, the preferable protein extraction methods were related to the protein precipitation using an alkaline, acid, or salted solution, followed by a specific filtration and drying technique (Table 1).

**Table 1:** Patents that use seeds and/or nuts as preferable raw material.

#	Preferable raw material	Publication number	Origin	Beverage type	Presentation form	Protein extraction methods and technologies applied	Ref.
1	Camellia seed	CN105053506 (A)	China	Protein-rich beverage	Powder	- Low temperature basic/acid protein precipitation; - Spray drying.	^79^
2	Ginkgo biloba seed	CN104543328 (A)	China	Beverage (without specification), carbonated beverage, alcoholic beverage	Powder or liquid	- Culture and fermentation; - Vacuum concentration; - Drying and milling.	^80^
3	Grape seed	US10335446 (B2); US2019000910 (A1)	United States of America	Juice	Powder or liquid	- Grinding pomace, centrifuge, and powder.	^81^
4	Hempseed	CN110250276 (A)	China	Protein-rich beverage	Liquid	- Formula composition.	^82^
5	Hempseed	WO2019213757 (A1)	World Intellectual Property Organization	Sports drink, infant formula	Powder or liquid	- Alkalinization followed by isoelectric protein precipitation; - Ultrafiltering and microencapsulation.	^83^
6	Hempseed	US2015079235 (A1)	United States of America	Milk analog, juice, infant formula	Powder or liquid	- Formula composition.	^84^
7	Hempseed	CN108522780 (A)	China	Beverage (without specification)	Powder	- Low-temperature physical squeezing extraction; - Physical superfine grinding.	^85^
8	Hempseed	CN110150391 (A)	China	Sport drink	Liquid	- Basic/acid protein precipitation; - Formula composition.	^86^
9	Hempseed	BR112015001964 (A2)	Brazil	Sport drink, protein-rich beverage, carbonated beverage, acid beverage	Powder or liquid	- Calcium salt protein precipitation; - Drying and diafiltration.	^87^
10	Moringa oleifera seed	CN110856518 (A)	China	Functional beverage	Liquid	- Ethanol and aqueous extraction; - Vacuum drying.	^88^
11	Pepper seed	CN110463818 (A)	China	Protein-rich beverage	Powder	- Alkaline protein precipitation; - Culture and fermentation; - Enzymolysis; - Spray drying.	^89^
12	Physalis seed	CN110432333 (A)	China	Beverage (without specification)	Powder	- Milling and baking the seeds.	^90^
13	Pumpkin seed	RU2590736 (C1)	Russian Federation	Milk analog	Powder	- Natural antibiotic solution maceration; - Drying and milling.	^91^
14	Pumpkin seed	BRPI0110757 (B1)	Brazil	Beverage (without specification)	Powder or liquid	- Formula composition.	^92^
15	Rapeseed	AR097442 (A2)	Argentina	Sports drink, protein-rich beverage	Powder	- Basic/acid protein precipitation.	^93^
16	Rapeseed	BRPI0308380 (B1)	Brazil	Protein-rich beverage, fermented beverage/yogurt analog	Powder	- Drying the supernatant of a protein micelles dispersion	^94^
17	Rapeseed	BR112012008321 (A2)	Brazil	Sports drink, carbonated beverage, juice, alcoholic beverage	Powder or liquid	- Supernatant heat treatment or isoelectric precipitation extraction.	^95^
18	Rapeseed	US2020154732 (A1)	United States of America	Protein-rich beverage	Powder	- Basic/acid protein precipitation.	^96^
19	Rapeseed	CN107873944 (A)	China	Functional beverage	Powder	- Enzymolysis; - Spray drying.	^97^
20	Rapeseed	WO2019234137 (A1)	World Intellectual Property Organization	Protein-rich beverage	Powder	- Basic/acid protein precipitation; - Formula composition.	^98^
21	Rapeseed	BRPI0913429 (A2)	Brazil	Carbonated beverage, sports drink, juice, protein-rich beverage	Powder or liquid	- Supernatant heat treatment or isoelectric protein precipitation.	^99^
22	Rapeseed	BRPI0917301 (A2)	Brazil	Protein-rich beverage	Powder	- Basic/acid protein precipitation.	^100^
23	Rapeseed	BRPI0917304 (A2)	Brazil	Protein-rich beverage, carbonated beverage, sport drink	Powder	- Calcium salt protein precipitation; - Acidic protein precipitation; - Diafiltration and drying.	^101^
24	Rapeseed	PL2323499 (T3)	Poland	Protein-rich beverage, acid beverage, carbonated beverage, sport drink	Powder	- Calcium salt protein precipitation; - Acidic protein precipitation; - Diafiltration and drying.	^102^
25	Rapeseed	BRPI1012171 (A2); BRPI1012171 (B1)	Brazil	Protein-rich beverage, acid beverage, carbonated beverage, sport drink	Powder	- Calcium salt protein precipitation; - Acidic protein precipitation; - Diafiltration and drying.	^103^
26	Rapeseed	BRPI0915489 (A2); BRPI0915489 (B1)	Brazil	Protein-rich beverage, carbonated beverage, acid beverage	Powder or liquid	- Calcium salt protein precipitation; - Acidic protein precipitation; - Diafiltration and drying.	^104^
27	Rapeseed	BRPI0917295 (A2)	Brazil	Sports drink, protein-rich beverage, carbonated beverage, acid beverage	Powder	- Calcium salt protein precipitation; - Acidic protein precipitation; - Diafiltration and drying.	^105^
28	Rapeseed	EP3481220 (A1)	European Patent Office	Beverage (without specification), fermented beverage/yogurt analog	Powder	- Basic/acid protein precipitation; - Drying.	^106^
29	Rapeseed	WO2019110556 (A1)	World Intellectual Property Organization	Protein-rich beverage, fermented beverage/yogurt analog, juice, milk analog	Powder	- Basic/acid protein precipitation; - Drying.	^107^
30	Rapeseed, mustard seed, broccoli seed, linseed, cottonseed, hempseed, safflower seed, or sesame seed	HUE033952 (T2)	Hungary	Protein-rich beverage, carbonated beverage, sport drink, milk analog, juice, infant formula	Powder	- Organic solvent or aqueous extraction; - Basic/acid protein precipitation; - Drying.	^108^
31	Rapeseed, mustard seed, broccoli seed, linseed, cottonseed, hempseed, safflower seed, sesame seed	PL2498620 (T3)	Poland	Milk analog, protein-rich beverage, carbonated beverage, sports drink, juice, infant formula	Powder	- Organic solvent or aqueous extraction; - Low g-force centrifugation.	^109^
32	Samara seed	CN104543326 (A)	China	Beverage (without specification)	Powder	- Supercritical carbon dioxide extraction.	^110^
33	Sesame seed	CN109430515 (A)	China	Fermented beverage/yogurt analog	Powder or liquid	- Alkaline protein precipitation; - Fermentation; - Drying.	^111^
34	Sesame seed	CN110810687 (A)	China	Beverage (without specification)	Liquid	- Enzymolysis; - Centrifugation.	^112^
35	Snake gourd seed	CN105192244 (A)	China	Beverage (without specification)	Powder or liquid	- Cold-pressed technique; - Enzymolysis; - Centrifugation; - Ultrafiltration; - Concentration and desiccation.	^113^
36	Sunflower seed	HUE029430 (T2)	Hungary	Milk analog	Powder	- Heated pressed technique.	^114^
37	Sunflower seed	CN109354574 (A)	China	Milk analog	Powder	- Supercritical carbon dioxide extraction; - Ethanol extraction; - Microwave extraction; - Ultrasonic extraction; - Basic/acid protein precipitation; - Spray drying.	^115^
38	Sunflower seed and/or cottonseed and/or rapeseed and/or coconut	CN110678083 (A)	China	Beverage (without specification)	Powder or liquid	- Formula composition.	^116^
39	Sunflower seed or rapeseed	RU2538147 (C1)	Russian Federation	Beverage (without specification)	Powder	- Basic/acid protein precipitation; - Drying and granulation.	^117^
40	Thistle seed	CN109266432 (A)	China	Beverage (without specification)	Powder or liquid	- Enzymolysis; - Centrifugation.	^118^
41	Walnut	CN209711374 (U)	China	Beverage (without specification)	Powder	- Alkaline protein precipitation; - Centrifugation; - Spray drying.	^119^

Based on the results, rapeseed was the most used seed, since it was the preferable raw material in 25 patents (Table 1). Rapeseed ranks second position of the major oilseed cultivated worldwide, it just loses its position to soybean^30^. Rapeseed and “canola” are the common names given to species *Brassica napus* L., *Brassica rapa* L., and *Brassica juncea* L; however, the name “canola” only refers to rapeseed varieties presenting less than 2% erucic acid and meal accounting for less than 30 μmol/g total glucosinolates. The crude protein in rapeseed meals ranges from 35 to 40%. At least 40 different protein fractions can be found in this total, but cruciferin, napin, and oleosin are the most investigated ones^5^. Cruciferin and napin are the main storage proteins, they account for 60 and 20% of the protein content in rapeseeds, respectively^26^. Oleosin presents structural proteins associated with oil components^31^. Although rapeseed has lower sulfur-amino acid levels^5^, canola meal has better amino acid profile than soybean. Nevertheless, canola proteins present many antinutritional factors, as among them glucosinolates, phenolics, phytates, as well as a large amount of fibers capable of compromising digestibility, color, taste, and physicochemical properties^29^. This finding explains some authors’ interest in patenting inventions that can solve these problem. Patents WO2019110556 (A1) and EP3481220 (A1) explain how to isolate inherently sweet rapeseed proteins presenting low content of antinutritional factors and high solubility, which can be used to reduce the amount of sucrose in beverages, and simultaneously increase protein content in the product. Likewise, BRPI0917295 (A2) provides rapeseed protein isolate that is soluble and stable not only in acid pH but also at higher temperatures. At the moment, Burcon^®^ is the only manufacturer of high-purity canola protein in the world^32,33^, despite the potential of this raw material.

Hemp (*Cannabis sativa* L.) was the preferable raw material in 11 patents (Table 1). It is mainly cultivated for the industrial use of its seeds, fibers, oil, and meal^34^. At the moment, hemp is at the mainstream, since it is a sustainable crop that does not require fertilizers, herbicides, and pesticides^35^. Hemp can be legally cultivated for industrial purposes in most countries worldwide, when it has up to 0.3% of its main psychoactive compound: delta-9-tetrahydrocannabinol (THC)^26^. Hemp seeds are rich in phytosterols (ω-3 and ω-6) and have ~25% of proteins in its dry weight^35^. Approximately 65% of the total hemp protein comprises a single globular storage protein: edestin; on the other hand, the albumin fraction consists of 25% of storage proteins^26^. Although it has low lysine content^15^, hempseed protein contains all the essential amino acids and accounts for low amount of antinutritional factors, such as trypsin inhibitor; therefore, it presents high-degree digestibility^35^. Hempseed protein can be appropriate for athletes and infants, for example, given their quality. Patent BR112015001964 (A2) provides a hempseed protein that is completely soluble in acid beverages (lower than 4.4 pH), without the need of stabilizers or additives. Thus, it can be used for protein fortification in soft and sports drinks, as well as in other aqueous systems. Similarly, CN110150391 (A) disclosed a canned beverage for athletes made with hempseed albumins, which is even better absorbed by the organism than the whole hempseed protein. US2015079235 (A1) proposed hempseed protein using as the main ingredient in an alternative infant formula, which can be either powdered or liquid. Currently, Axiom Foods^®^ and Good Hemp^®^, which offer products with 58 and 85% purity, respectively, are among the hempseed protein suppliers in the world^36,37^. Also, Manitoba Harvest^®^ and LeanHemp^®^ are brands focusing on the hempseed protein supplement segment^38,39^.

Ten patents used Sunflower (*Helianthus annuus* L) as preferable raw material (Table 1). This species is one of the most cultivated oilseed crops^40^; its seed meal can reach 40% protein content when it is mechanically extracted, and 50% protein content when the oil is extracted with organic solvents^5^. Approximately 85% of the total protein content in sunflower seeds is found in the form of storage proteins (11S helianthinin globulin and 2S albumins)^26^. Globulin content varies from 40 to 90%, whereas albumin rate ranges from 10 to 30% of the total protein found in seeds. It is possible observing differences in the ratio of these protein fractions since three different sunflower seed types are majorly cultivated—they are used for oil production, human and pet consumption, and ornamentation^5^. Despite its low lysine content, sunflower meal remains a good protein source, given its high digestibility and smaller number of antinutritional compounds^41^. Although sunflower seed proteins have low functionality, such a feature is closely linked to denaturation caused by heating at oil extraction, rather than to native proteins themselves. Moreover, phenolic compounds found in sunflower seeds, such as chlorogenic acid, can crosslink with proteins and change its color, functionality, and bioavailability. However, chlorogenic acid is also beneficial for human health^40^ and can be desirable when it is properly extracted from sunflower seed meal. Patent CN109354574 (A) improved a method by using supercritical carbon dioxide extraction to prepare chlorogenic acid and protein powder from sunflower seeds, in separate. This powder is an interesting raw material for making artificial milk and beverages due to its soft flavor. Similarly, FI128029 (B) addresses a sunflower seed protein with enhanced solubility and low phytate content. Its powder is a suitable ingredient for dairy alternatives. Following functionality improvements, BR112018070543 (A2) used enzymatic treatment, heating, fermentation, and pressure treatment to increase the techno-functional and organoleptic properties of sunflower seed protein, which is useful as a beverages’ supplement. Presently, Biotechnologies^®^ is the only world sunflower seed protein producer whose product has more than 80% purity^42^; However, it is possible finding other suppliers whose products reach about 50% protein content, among them one finds Austrade Inc^®^ and ETChem^[®43,44^.

Based on the results, cottonseed was the preferable raw material in seven patents (Table 1). Fiber is the most common product made out of cotton; however, cottonseed has great potential as protein source, for both animal and human consumption^45^. Cottonseed meal has ~30 to 50% total protein^46^. Arginine has the highest content (22–34%) of essential amino acids, whereas cysteine accounts for the lowest one (1–2%)^47^. Cottonseed meal is a good protein source for ruminants, but it is not appropriate without treatment for monogastric animals, including humans, who do not tolerate high fiber contents and antinutritive compounds, such as gossypol, phytin, and cyclopropane fatty acids^46^. In total, 80% of defatted cottonseed is used as substrate for edible mushroom crops or as fertilizer, but only 5 to 10% of it is used for animal feeding purposes, a fact that causes huge protein waste^48^. The appropriate extraction of these proteins from the meal is a feasible option to solve this problem. Patents HUE033952 (T2) and PL2498620 (T3) disclosed enhanced methods to produce high-quality protein concentrates and isolates from oilseed meals, including cottonseed, which has incredibly low fiber content and antinutritional factors. These concentrates and isolates are useful as powdered milk analog or to increase protein content of juices, soft drinks, sports drinks, and beverages in general. RU2018129466 (A) disclosed a method to extract cottonseed proteins that can fortify beverages as well. When it is compared to other patents, it also has the advantage of not presenting beany or grassy flavor notes. Nevertheless, so far, cottonseed protein suppliers in the market only provide to animal feeding, they still do not focus on human nutrition.

### Legumes and pulses

Legumes are the most consumed staple food after cereals, since they are a low-priced source of nutrients, mainly of proteins; therefore, they are seen as “meat for poor men”^14,15^. In botanical terms, legumes belong to the family *Leguminosae*, which also comprises pulses. All pulses are legumes, although not all legumes are pulses. Based on the Food and Agriculture Organization definition, pulses are only dried edible leguminous presenting low oil content. Consequently, legumes harvested in green forms, such as green beans and green peas, are not included in this group. Furthermore, soybeans and peanuts, which are oil-rich, are also excluded from it. Lastly, crops used for sowing purposes, such as alfalfa and clover, are not pulses^49^. Pulses are good energy, fiber, vitamin, mineral, and bioactive compound sources, and besides, ~30% of pulses’ dry bases are proteins^50^. In addition to their nutritional importance, pulses have the good nitrogen-fixing capacity and are beneficial to soil fertility and the environment^51^. Some native pulses and legumes’ proteins are promising to the beverage industry due to their functional features, such as solubility, emulsifying, and foaming proprieties^50^. However, depending on the species and on the protein extraction method, beverages made with these proteins also show beany flavor, bitter taste, or sandy mouthfeel^4,14,15^ —these factors limit their acceptance by consumers. Another disadvantage of these proteins lies in the fact that different procedures must be followed in order to reduce antinutritional factors and nonprotein compounds from pulses and legumes. Although, isolate proteins extracted from legumes and pulses remain in the mainstream of researchers and patent applicants as they are an economical, ecological, nutritive, and gluten-free raw material used to enrich products^50^. Enzymolysis and protein precipitation using an alkaline, acid, or salted solution were the preferable protein extraction methods applied, commonly followed by a specific filtration and drying technique (Table 2).

**Table 2:** Patents that use legumes and/or pulses as preferable raw material.

#	Preferable raw material	Publication number	Origin	Beverage type	Presentation form	Protein extraction methods and technologies applied	Ref.
1	Alfalfa, lentil, bean, pea, lupine, locust bean, peanut, tamarind, wisteria, chickpea, fenugreek, or combinations thereof	US2019000112 (A1)	United States of America	Milk analog, fermented beverage/yogurt analog, protein-rich beverage, sports drink, infant formula	Powder or liquid	- Basic/acid protein precipitation; - Salt protein precipitation; - Formula composition.	^120^
2	Bean and/or pea and/or chickpea and/or lentil	EP3573471 (A1)	European Patent Office	Milk analog	Powder	- Enzymolysis; - Centrifugation; - Spray drying.	^121^
3	Black bean or pea	US2019133150 (A1)	United States of America	Protein-rich beverage	Powder or liquid	- Freezing raw material; - Grinding.	^122^
4	Black-eyed pea	US2019239535 (A1)	United States of America	Acid beverage, carbonated beverage, protein-rich beverage, functional beverage, fermented beverage/yogurt analog	Powder or liquid	- Wet grinding; - Basic/acid protein precipitation.	^123^
5	Broad bean	WO2020051622 (A1)	World Intellectual Property Organization	Protein-rich beverage	Powder	- Milling; - Hydrating; - Filtrating; - Pasteurizing milk-like fluid.	^124^
6	Chickpea	JP2019520082 (A)	Japan	Milk analog	Powder	- Basic/acid protein precipitation; - Ultrafiltration; - Evaporation.	^125^
7	Hyacinth bean	CN104774271 (A); CN104774271 (B)	China	Beverage (without specification)	Powder	- Acidic protein precipitation; - Enzymolysis; - Centrifugation; - Drying.	^126^
8	Kidney bean, red bean, and peanut	KR102092404 (B1); KR20200013276 (A)	Republic of Korea	Milk analog	Liquid	- Formula composition.	^127^
9	Lentil	EP3209141 (A1); EP3209141 (B1)	European Patent Office	Beverage (without specification)	Powder or liquid	- Formula composition.	^128^
10	Lentil, chickpea, pea, or bean	WO2020061698 (A1)	World Intellectual Property Organization	Milk analog	Powder or liquid	- Basic/acid protein precipitation; - Heating; - Drying.	^129^
11	Lentil, chickpea, pea, or bean	BR112015007140 (A2)	Brazil	Sports drink, protein-rich beverage, acid beverage, carbonated beverage, milk analog	Powder	- Calcium salt protein precipitation; - Diafiltration and drying.	^130^
12	Lentil, chickpea, pea, or bean	KR20190087654 (A)	Republic of Korea	Protein-rich beverage	Powder	- Calcium salt protein precipitation; - Drying.	^131^
13	Lentil, chickpea, pea, or bean	EP3586644 (A1)	European Patent Office	Protein-rich beverage, carbonated beverage, milk analog	Powder or liquid	- Calcium salt protein precipitation; - Acidic protein precipitation; - Diafiltration and drying.	^132^
14	Lentil, chickpea, pea, or bean	JP2018110600 (A)	Japan	Protein-rich beverage, acid beverage, carbonated beverage, sport drink	Does not specify	- Calcium salt protein precipitation; - Acidic protein precipitation; - Diafiltration and drying.	^133^
15	Lupine	CA2953644 (A1)	Canada	Milk analog, fermented beverage/yogurt analog	Liquid	- Supercritical carbon dioxide extraction.	^134^
16	Lupine	RU2555528 (C1)	Russian Federation	Fermented beverage/yogurt analog	Liquid	- Acidic protein precipitation; - Enzymolysis; - Pasteurizing.	^135^
17	Mung bean	US2019191735 (A1)	United States of America	Protein-rich beverage, milk analog, fermented beverage/yogurt analog	Powder or liquid	- Basic/acid protein precipitation; - Ultrafiltration.	^136^
18	Mung bean	JP6521257 (B2); JPWO2015105138 (A1)	Japan	Protein-rich beverage	Powder	- Formula composition.	^137^
19	Mung bean	JP6332266 (B2); JPWO2014156549 (A1)	Japan	Fermented beverage/yogurt analog	Gel	- Formula composition.	^138^
20	Mung bean	CN106261782 (A)	China	Fermented beverage/yogurt analog	Powder	- Fermentation; - Drying.	^139^
21	Mung bean and/or chickpea and/or pea	CA2982280 (A1)	Canada	Milk analog	Liquid	- Formula composition.	^140^
22	Pea	MX2018012893 (A)	Mexico	Fermented beverage/yogurt analog	Liquid	- Acidic protein precipitation; - Fermentation.	^141^
23	Pea	CA3067593 (A1)	Canada	Sports drink, carbonated beverage, juice, alcoholic beverage	Powder or liquid	- Formula composition.	^142^
24	Pea	WO2020007940 (A1)	World Intellectual Property Organization	Sport drink, protein-rich beverage, infant formula	Powder	- Formula composition.	^143^
25	Pea	FR3047151 (A1)	France	Protein-rich beverage, sport drink	Powder or liquid	- Formula composition.	^144^
26	Pea	BR112019022289 (A2)	Brazil	Protein-rich beverage, milk analog	Liquid	- Basic/acid protein precipitation; - Drying.	^145^
27	Pea	CN107668314 (A)	China	Functional beverage	Powder	- Enzymolysis; - Centrifugation; - Spray drying.	^146^
28	Pea	US2016316785 (A1)	United States of America	Fermented beverage/yogurt analog, juice, alcoholic beverage	Powder	- Acidic protein precipitation; - Heating.	^147^
29	Pea	FR3070831 (A1)	France	Sports drink, protein-rich beverage, infant formula	Liquid	- Formula composition.	^148^
30	Pea	CN109907156 (A)	China	Functional beverage	Powder	- Enzymolysis.	^149^
31	Pea	MX2018010758 (A)	Mexico	Fermented beverage/yogurt analog, coffee whitener, protein-rich beverage	Powder or liquid	- Basic/acid protein precipitation; - Pasteurizing; - Spay drying.	^150^
32	Pea	CO2020004049 (A2)	Colombia	Beverage (without specification)	Powder	- Isoelectric protein precipitation; - Ultrafiltration.	^151^
33	Pea	CA3079976 (A1)	Canada	Protein-rich beverage, juice	Powder or liquid	- Enzymolysis.	^152^
34	Pea	US2019053517 (A1)	United States of America	Beverage (without specification)	Powder	- Enzymolysis.	^153^
35	Pea	CN109566747 (A)	China	Fermented beverage/yogurt analog	Liquid	- Enzymolysis; - Basic/acid protein precipitation; - Fermentation.	^154^
36	Pea	BR112019018323 (A2)	Brazil	Acid beverage	Powder	- Acidic protein precipitation.	^155^
37	Pea	ES2676925 (T3)	Spain	Sport drink	Powder	- Cetrifugation; - Ultrafiltration.	^156^
38	Pea	CN109907155 (A)	China	Functional beverage	Powder	- Basic/acid protein precipitation; - Enzymolysis; - Spray drying.	^157^
39	Pea	EA030803 (B1); EA201691510 (A1)	Eurasian Patent Organisation	Carbonated beverage, juice	Does not specify	- Adsorbent resin extraction.	^158^
40	Pea	WO2020109741 (A1)	World Intellectual Property Organisation	Acid beverage, carbonated beverage	Powder	- Enzymolysis; - Drying.	^159^
41	Pea	US2020100524 (A1)	United States of America	Fermented beverage/yogurt analog, milk analog, sports drink, carbonated beverage, acid beverage, protein-rich beverage	Powder	- Basic/acid protein precipitation; - Enzymolysis; - Ultrafiltration.	^160^
42	Pea	CA3008464 (A1)	Canada	Milk analog, fermented beverage/yogurt analog	Powder or liquid	- Acidic protein precipitation. - Enzymolysis; - Ultrafiltration.	^161^
43	Pea	CN111066944 (A)	China	Beverage (without specification)	Does not specify	- Formula composition.	^162^
44	Pea, lentil, chickpea, peanut, pinto bean, Great Northern bean, navy bean, red bean, black bean, kidney bean, broad bean, baby lima bean, pink bean, mayocoba bean, black-eyed pea, cranberry bean, white bean, rice bean, butter bean, or combinations thereof	US2017087209 (A1)	United States of America	Iron-rich beverage	Powder or liquid	- Salt protein precipitation; - Enzymolysis; - Filtration.	^163^
45	Peanut	CN106260496 (A)	China	Beverage (without specification)	Powder	- Basic/acid protein precipitation; - Drying.	^164^
46	Peanut	CN106367196 (A); CN106367196 (B)	China	Beverage (without specification)	Powder	- Formula composition.	^165^
47	Peanut	CN107927319 (A)	China	Juice	Liquid	- Enzymolysis.	^166^
48	Peanut	CN110140801 (A)	China	Juice	Powder or liquid	- Enzymolysis; - Spray drying.	^167^
49	Peanut	US2020170279 (A1)	United States of America	Beverage (without specification)	Does not specify	- Basic/acid protein precipitation; - Heating; - Enzymolysis; - Centrifugation.	^168^
50	Peanut	CN106333057 (A)	China	Milk analog	Powder	- Low temperature basic/acid protein precipitation; - Spray drying.	^169^
51	Peanut	FR3072002 (A1); FR3072002 (B1)	France	Fermented beverage/yogurt analog	Gel	- Enzymolysis.	^170^

Pea was the most used raw material in the herein assessed patents, it was the preferable raw material in 43 patents (Table 2). Pea is an ancient crop widely grown in different countries^50^. At the moment, pea stands out for its properties similar to those of soybean protein; therefore, it presents great potential to replace this protein, since soybean is often associated as allergenic^13^. Emulsification, foaming, and gelling are some of these properties, for instance^14^. Pea has 23 to 31% total protein, and 50 to 85% are globulins (legumin, vicilin, and convicilin), while 15 to 25% are albumins^50^. Arginine, phenylalanine, leucine, and isoleucine are among the essential amino acids commonly found in the globulin proteins, whereas tryptophan, lysine, threonine, cysteine, and methionine are found in the albumin ones^52^. Overall, the amino acid profile is well-balanced and the high lysine content is notable^13^. Even so, the bitter taste intrinsic to pea flour produced through dry milling can be transferred to final products. Patent CA3067593 (A1) disclosed methods to produce pea flour, concentrate or isolate with low nonvolatile flavor compound contents, specifically bound saponin, in order to solve this problem. This process consists of steam cooking and drying a raw pea slurry to produce a bitterless flour that can be used in beverages. Similarly, FR3070831 (A1) used sodium citrate and heating to partially or fully reduce the bitterness of pea protein compositions. Fermentation can also improve flavor; therefore, yogurt-like products are a good option to be made of pea protein, as proposed by CN109566747 (A). Pea protein has been consolidated in the market by world suppliers such as Roquette^®^, Burcon^®^, Axiom Foods^®^, AMCO Proteins^®^, Ingredion^®^, Cargill^®^, and Farbest^[®53–59^. Pea protein milk and yogurt are already available and provided by Ripple^®^ and Siggi’s^®^, respectively^60,61^.

Based on the results, bean was the preferable raw material in 21 patents (Table 2). Common beans belonging to the family *Leguminosae* and to genus *Phaseolus* account for one-third of global pulses production^62^. There are more than 14,000 known different common bean cultivars. Peculiarities of local culture, agriculture, and history influence the mostly consumed beans. Variations on beans’ size, shape, and colors can occur, as well as on their composition and physicochemical features, but these differences tend to be nonsignificant^63^. Overall, dry beans are an important source of proteins—its protein rate ranges from 16 to 33%^50^. Albumins account for up to 10–30% of total protein content on dry weight basis, whereas globulins account for 45–70% of it^52^. Phaseolin and lectin are other important protein fractions found in beans^50^. Although the protein content in beans is significant, it shows some drawbacks, since beans have methionine as limiting amino acid and antinutritional factors are observed in them^62^. These factors can be removed from pulses through a simple fermentation process; consequently, it increases the quality of pulses’ proteins^15^. Patent JPWO2014156549 (A1) disclosed methods to produce a gel by adding alkali metal ions to mung bean protein and by fermenting it. The produced composition can be used as yogurt analog. Similarly, CN106261782 (A) used biological mung bean fermentation to separate protein, starch, fiber, and slurry, and to provide different products, such as instant tablet beverage and fermented drink. Once more, to reduce antinutritional factors, KR20190087654 (A) developed a bean protein presenting low phytic acid content by using calcium salt to precipitate it. At the moment, It is possible finding 80% purity mung bean and fava bean protein suppliers, as among them one finds ETProtein^®^ and Atura^®^, respectively^64,65^. The brand Green Boy^®^ also offers powdered proteins extracted from these same bean species^66,67^. Nancy’s^®^ produces yogurts with different flavors, all made of fava bean protein^68^.

In total, 18 patents were related to peanut’s use as preferable raw material (Table 2). Peanut accounts for 11% of the global protein supply^69^; it has been gaining more popularity due to its resistance to climate changes^8^, as well as because it is a cheap source of high-quality protein. Deffated meal, which is a by-product by the peanut industry, has ~50–55% protein and balanced amino acid profile^70^; therefore, it can be easily used as raw material to produce protein isolates^71^. Peanut proteins are often classified as albumins and globulins. Arachin and conarachin are the main globulins, and both of them are rich in lysine, tryptophan, tyrosine, and phenylalanine^69^. Peanut protein is often nutritionally comparable to animal protein^72^, mainly after fermentation, since this process increases its l-lysine, l-methionine, and l-tryptophan contents^1^. However, its allergenicity^69^ and poor functional proprieties hamper peanut protein use by the food industry^70^. Different treatments, such as chemical, physical, or enzymatic methods, have been applied to expand peanut protein functionality. Patent CN106260496 (A) disclosed an improved ultrasonic treatment applicable to peanut protein that provides better solubility, higher yield, and efficiency, as well as environmental protection, when compared to the state of the art. FR3072002 (A1) addresses a stable gel that can be produced by improving peanut protein solubility followed by adding transglutaminase to it. This gel is highly useful to produce yogurts. According to CN106367196 (A), it is possible obtain peanut protein that presents stability in beverages by preventing its denaturation during the extraction process. High-purity peanut protein isolates or concentrates are not in the market yet; however, deffated peanut powder is already provided by ETChem^®^ and Peanut Butter & Co^®^, for example^73,74^.

Based on the results, chickpea was the preferable raw material in 12 patents (Table 2). It is the second mostly produced legume worldwide and its protein amount ranges from 15 to 30%. Globulins (53 to 60%) and glutelins (19 to 25%) are the main protein fractions in chickpea, but albumins (8 to 12%), and prolamins (3 to 7%) are also relevant in it^50^. Although chickpea lacks sulfured amino acids^52^, it has low content of antinutritional factors and, consequently, high bioavailability of other essential amino acids^14^. Blend chickpea with other pulses that do not lack sulfured amino acids is an interesting solution to its limiting amino acids issue, so the quality of the protein in the final product increases significantly. Patent CA2982280 (A1) provided a method to produce an alternative to milk by using chickpea and/or pea protein as main ingredients. Moreover, the disclosed product has a smooth mouthfeel and does not necessarily need gum, emulsifier, or starch to be added to the formulation. On the other hand, EP3586644 (A1) and WO2020061698 (A1) focused on the production of pulse proteins, including chickpea. These proteins are stable both in hot and acid aqueous environments. Among chickpea proteins available in the market, finds the ones by Innovopro^®^ and Atura^®^, which sell products with 70 and 80% protein contents, respectively^75,76^. The brand Green Boy^®^ also has chickpea and other different plant-based proteins in its portfolio.

### Other mixes

The group “other mixes” regards patents that used either seeds, nuts, legumes, pulses, cereals, seaweeds, fungus, or combinations of these elements. These combinations include blended products to reach a better amino acid score and to optimize the functional and sensorial features of final product. Such an outcome can be obtained through the interaction of proteins from different plant sources (Table 3).

**Table 3:** Patents that use other mixes as preferable raw material.

#	Preferable raw material	Publication number	Origin	Beverage type	Presentation form	Protein extraction methods and technologies applied	Ref.
1	Alfalfa and/or wheat	WO2019150144 (A1)	World Intellectual Property Organization	Sport drink	Powder	- Heating - Microwave extraction; - Filtration.	^171^
2	Almond, cashew nut, Brazil nut, coconut, hazelnut, macadamia, peanut, walnut, or pistachio	CN110742128 (A)	China	Fermented beverage/yogurt analog	Liquid	- Enzymolysis; - Centrifugation; - Fermentation.	^172^
3	Cashew nut and/or almond and/or peanut and/or pea and/or oat and/or wheat and/or quinoa	EP3429366 (A1)	European Patent Office	Milk analog	Liquid	- Formula composition.	^173^
4	Chia seed and/or pumpkin seed and/or hempseed and/or almond and/or macadamia and/or lentil and/or pea and/or chickpea and/or mung bean and/or rice	US2019225645 (A1)	United States of America	Sports drink, protein-rich beverage, infant formula	Does not specify	- Formula composition.	^174^
5	Does not specify	CN110637916 (A)	China	Fermented beverage/yogurt analog	Powder	- Fermentation.	^175^
6	Does not specify (Legume and/or seed and/or cereal and/or seaweed)	JP2017521498 (A)	Japan	Carbonated beverage, juice, milk analog, sports drink, functional beverage, fermented beverage/yogurt analog, alcoholic beverage	Powder or liquid	- Heating; - Enzymolysis; - Nanofiltration; - Spray drying.	^176^
7	Does not specify (Legume and/or seed and/or seaweed and/or fungus	EP3634146 (A1)	European Patent Office	Protein-rich beverage, milk analog, coffee whitener, fermented beverage/yogurt analog, sport drink, juice	Powder or liquid	- Ultrasonication; - Filtration.	^177^
8	Pea and coconut	US2019307143 (A1)	United States of America	Coffee whitener	Liquid	- Isoelectric protein precipitation or enzymolysis.	^178^
9	Pea and rice	CN111227101 (A)	China	Beverage (without specification)	Powder	- Formula composition.	^179^
10	Pea and/ or lupine and/or broad bean and/or rapeseed and/or turnip seed and/or sunflower seed	FI128029 (B)	Finland	Milk analog	Does not specify	- Formula composition.	^180^
11	Pea and/or rice and/or oat	FR3085826 (A3)	France	Beverage (without specification)	Powder	- Formula composition.	^181^
12	Pea and/or wheat and/or oat	WO2019228957 (A1)	World Intellectual Property Organization	Beverage (without specification), carbonated beverage, juice, alcoholic beverage	Powder or liquid	- Formula composition.	^182^
13	Pea or lupine or bean or chickpea or lentil or peanut or sunflower seed or rapeseed or camelina seed or linseed	BR112018070543 (A2)	Brazil	Milk analog, fermented beverage/yogurt analog	Powder	- Organic solvent extraction; - Enzymolysis; - Heating; - Fermentation.	^183^
14	Pea or rice	BR112019019992 (A2)	Brazil	Infant formula	Powder or liquid	- Enzymolysis; - Centrifugation; - Nanofiltration; - Pasteurizing; - Spay drying.	^184^
15	Pea, walnut, almond, cashew nut, hempseed, or rice	US2020060310 (A1)	United States of America	Milk analog, fermented beverage/yogurt analog, protein-rich beverage	Powder	- Formula composition.	^185^
16	Peanut and moringa oleifera seed	FR3019004 (A1); FR3019004 (B1)	France	Juice	Powder	- Enzymolysis; - Heating; - Acidic protein precipitation; - Drying.	^186^
17	Peanut, rapeseed, sesame seed, linseed, sunflower seed, cottonseed, safflower seed, perilla seed, castor bean coconut, cocoa seed, almond, tung seed, sesame seed, primula seed, hazelnut, pumpkin seed, walnut, grape seed, sea buckthorn seed, tomato seed pumpkin seed, macadamia, oat, corn, rice, wheat, seaweed, or combinations thereof	CN107125430 (A)	China	Fermented beverage/yogurt analog	Powder or liquid	- Enzymolysis.	^187^
18	Rapeseed, sunflower seed, hempseed, safflower seed, cottonseed, linseed, sesame seed, mustard seed, peanut	RU2018129466 (A)	Russian Federation	Protein-rich beverage	Powder	- Acidic protein precipitation; - Diafiltration; - Drying.	^188^
19	Sunflower seed, cottonseed, rapeseed, coconut, peanut	WO2020028446 (A1)	World Intellectual Property Organization	Protein-rich beverage	Powder	- Formula composition.	^189^
20	Sunflower seed, cottonseed, rapeseed, coconut, peanut, locust bean, or combinations thereof	US10645950 (B2); US2020060308 (A1)	United States of America	Beverage (without specification)	Powder or liquid	- Formula composition.	^190^
21	Wheat and/or barley and/or corn and/or pea and/or rice	JP6059659 (B2); JPWO2013027813 (A1)	Japan	Carbonated beverage, alcoholic beverage	Does not specify	- Enzymolysis; - Fermentation.	^191^

FR3085826 (A3) disclosed a powder ready to be reconstituted in water or plant-based milk. This process uses proteins from peas, rice, oats, or from their mixes. Similarly, EP3429366 (A1) divulges a replacement for dairy milk, which is made of peanut protein—but it can also be made of cashew nut, almond, pea, oat, wheat, quinoa, or of combinations of these elements.

In regard to functional improvements, CN111227101 (A) provided an instant powdered beverage that can be composed of pea and rice proteins, which shows excellent solubility in hot water (≥85 °C). JPWO2013027813 (A1) presented a beer-like product with increased foam stability that was produced with protease, transglutaminase, and with one or more proteins from wheat, barley, corn, pea, or rice.

Focusing on sensorial aspects, WO2019228957 (A1) described a method to mask undesirable notes from beverages made of pea, wheat, or oat protein when ethyl cyclohexanoate was used as additive. EP3634146 (A1) suggested the application of ultrasound and membrane filtration processes in proteins from legumes, seeds, seaweeds, or fungus to obtain a clean taste and neutral color product that can be used to enrich a wide range of beverages.

A huge variety of blended products has been introduced in the market. The supplier Burcon^®^ developed a combination of pea and canola proteins, which is nutritionally and functionally better than when these proteins are used separated. The brand Orgain^®^ offers a blend of deffated peanut powder, pea protein, pumpkin seed protein, and almond protein^77^, while Manitoba Harvest^®^ commercializes a mix of pea and hemp proteins, which is ideal to be added to smoothies or nondairy milks^78^.

## Conclusion

Plant proteins extraction to enrich plant-based beverages has recently emerged as solution to lack of this nutrient in these products. However, some other factors, such as poor solubility, off-flavor notes, limiting amino acids, and anti-nutrients remains an issue. Chemical, physical, biological, and enzymatic treatments were reported in patents to solve these problems. Among them, acidification, basification, heating, membrane filtration, centrifugation, ultrasonication, fermentation, or combinations thereof were frequently applied, with the specificity and uniqueness methodology of each patent. Moreover, it has been comprehended that each step of industrial processes, since protein extraction to its incorporation to final products, have huge influence on their nutritional, sensorial, and functional features. Pea, rapeseed, bean, peanut, chickpea, hempseed, sunflower seed, and cottonseed were the most addressed raw materials in the herein assessed patents. Protein blends also emerged as simple alternatives to improve amino acid profile, as well as the functional and sensorial aspects of beverages. Expansion in the use of plant proteins to enrich multiple categories of beverages is another tendency observed. These beverages include sport drinks, carbonated beverages, or even juices, not limited to products that mimic dairy. Briefly, research and development based on plant-based protein have been playing important role in the production of beverages by fulfilling the existing demands from vegetarians, lactose intolerants, and cow milk allergic individuals, but also by turning these products into a suitable option for a wide range of other consumers, such as infants, elderly, athletes, or those who chose a healthier lifestyle. Thus, plant proteins are a promising ingredient to create high-quality beverages, ensuring animal welfare, reducing environmental impact, and guarantying food security if proper technology is applied to them.

## Data Availability

All data generated or analyzed during this study are included in this published article.

## References

[1] Tangyu M, Muller J, Bolten CJ, Wittmann C (2019). Fermentation of plant-based milk alternatives for improved flavour and nutritional value. Appl. Microbiol. Biotechnol..

[2] Aydar EF, Tutuncu S, Ozcelik B (2020). Plant-based milk substitutes: bioactive compounds, conventional and novel processes, bioavailability studies, and health effects. J. Funct. Foods.

[3] Wirnitzer, K. C. In *Therapeutic, Probiotic, and Unconventional Foods* Ch. 21 (eds Grumezescu, A. & Holban, A. M.) (Elsevier, 2018).

[4] Mäkinen OE, Wanhalinna V, Zannini E, Arendt EK (2015). Foods for special dietary needs: non-dairy plant-based milk substitutes and fermented dairy-type products. Crit. Rev. Food Sci. Nutr..

[5] Arrutia F, Binner E, Williams P, Waldron KW (2020). Oilseeds beyond oil: press cakes and meals supplying global protein requirements. Trends Food Sci. Technol..

[6] Sethi S, Tyagi SK, Anurag RK (2016). Plant-based milk alternatives an emerging segment of functional beverages: a review. J. Food Sci. Technol..

[7] Mintel Group LTDA. You heard it here first: the plant-based revolution. https://www.mintel.com/blog/food-market-news/you-heard-it-here-first-predicting-the-plant-based-revolution (2019).

[8] Jeske S, Zannini E, Arendt EK (2017). Past, present and future: the strength of plant-based dairy substitutes based on gluten-free raw materials. Food Res. Int..

[9] Banovic M (2018). Foods with increased protein content: a qualitative study on European consumer preferences and perceptions. Appetite.

[10] Mintel Group LTDA. US sales of dairy milk turn sour as non-dairy milk sales grow 9% in 2015. https://www.mintel.com/press-centre/food-and-drink/us-sales-of-dairy-milk-turn-sour-as-non-dairy-milk-sales-grow-9-in-2015 (2016).

[11] Vanga SK, Raghavan V (2018). How well do plant based alternatives fare nutritionally compared to cow’s milk?. J. Food Sci. Technol..

[12] Rincon L, Botelho RBA, de Alencar ER (2020). Development of novel plant-based milk based on chickpea and coconut. LWT.

[13] Graça C, Raymundo A, de Sousa I (2016). Rheology changes in oil-in-water emulsions stabilized by a complex system of animal and vegetable proteins induced by thermal processing. LWT Food Sci. Technol..

[14] Qamar S, Manrique YJ, Parekh H, Falconer JR (2020). Nuts, cereals, seeds and legumes proteins derived emulsifiers as a source of plant protein beverages: a review. Crit. Rev. Food Sci. Nutr..

[15] Schweiggert-Weisz U, Eisner P, Bader-Mittermaier S, Osen R (2020). Food proteins from plants and fungi. Curr. Opin. Food Sci..

[16] Gorissen SHM (2018). Protein content and amino acid composition of commercially available plant-based protein isolates. Amino Acids.

[17] Clifton PM (2011). Protein and coronary heart disease: the role of different protein sources. Curr. Atheroscler. Rep..

[18] Lin Y (2011). Plant and animal protein intake and its association with overweight and obesity among the Belgian population. Br. J. Nutr..

[19] Banno A (2019). Identification of a novel cholesterol-lowering dipeptide, phenylalanine-proline (FP), and its down-regulation of intestinal ABCA1 in hypercholesterolemic rats and Caco-2 cells. Sci. Rep..

[20] Chan-Zapata I, Sandoval-Castro C, Segura-Campos MR (2020). Proteins and peptides from vegetable food sources as therapeutic adjuvants for the type 2 diabetes mellitus. Crit. Rev. Food Sci. Nutr..

[21] Piovesana S (2018). Recent trends and analytical challenges in plant bioactive peptide separation, identification and validation. Anal. Bioanal. Chem..

[22] Montesano D, Gallo M, Blasi F, Cossignani L (2020). Biopeptides from vegetable proteins: new scientific evidences. Curr. Opin. Food Sci..

[23] Ferreira AA, Guimarães ER, Contador JC (2009). Patente como instrumento competitivo e como fonte de informação tecnológica. Gest.ão Produção.

[24] Al Kassiri M, Čorejová T (2015). Importance of patent and innovation in educational institutions. CBU Int. Conf. Proc..

[25] New Food Magazine. PBMAs are the plant-based proteins to watch out for. https://www.newfoodmagazine.com/article/128130/pbma/ (2020).

[26] Kotecka-Majchrzak K, Sumara A, Fornal E, Montowska M (2020). Oilseed proteins – Properties and application as a food ingredient. Trends Food Sci. Technol..

[27] USDA. Oilseeds: world markets and trade. https://downloads.usda.library.cornell.edu/usda-esmis/files/tx31qh68h/8w32r601z/1g05fb91f/oilseed-trade-05-10-2018.pdf (2018)

[28] Gomes S (2019). Microencapsulated Brazil nut (Bertholletia excelsa) cake extract powder as an added-value functional food ingredient. LWT.

[29] Tan SH, Mailer RJ, Blanchard CL, Agboola SO (2011). Canola proteins for human consumption: extraction, profile, and functional properties. J. Food Sci..

[30] Campbell L, Rempel CB, Wanasundara JPD (2016). Canola/rapeseed protein: future opportunities and directions—workshop proceedings of IRC 2015. Plants.

[31] Aider M, Barbana C (2011). Canola proteins: composition, extraction, functional properties, bioactivity, applications as a food ingredient and allergenicity - A practical and critical review. Trends Food Sci. Technol..

[32] Supertein. https://burcon.ca/products/canola-proteins/supertein/ (2021).

[33] Food in Canada. BurconCompany, merit functional foods, achieves first commercial production of canola protein. https://www.foodincanada.com/food-business/burconcompany-merit-functional-foods-achieves-first-commercial-production-of-canola-protein-148054/ (2021).

[34] Vonapartis E, Aubin MP, Seguin P, Mustafa AF, Charron JB (2015). Seed composition of ten industrial hemp cultivars approved for production in Canada. J. Food Compos. Anal..

[35] Mamone G, Picariello G, Ramondo A, Nicolai MA, Ferranti P (2019). Production, digestibility and allergenicity of hemp (Cannabis sativa L.) protein isolates. Food Res. Int..

[36] Axiom Foods. Cannatein® hemp hearts protein. http://axiomfoods.com/cannatein-hemp-protein/ (2021).

[37] Good Hemp Ingredients. White hemp seed protein isolate 85%. https://goodhemp-ingredients.com/ingredients/hemp-protein/white-hemp-seed-protein-isolate-85/ (2021).

[38] Manitoba Harvest. Hemp yeah! Max protein unsweetened. https://manitobaharvest.com/products/hemp-yeah-max-protein-unsweetened (2021).

[39] LeanHemp. Hemp protein is the best vegan protein. https://leanhemp.com/#1461725831070-43211078-316c (2021).

[40] González-Pérez, S. *Sunflower Proteins* (Elsevier, 2015).

[41] Murru M, Calvo CL (2020). Sunflower protein enrichment. Methods and potential applications. OCL.

[42] Bio Technologies LLC. Sunprotein. http://www.bio-t.pro/eng/products/food/sunprotein (2021).

[43] Austrade Inc.—Sunflower Protein Supplier. Heliaflor® sunflower proteins. https://www.austradeinc.com/heliaflor/ (2021).

[44] ETChem. Sunflower protein manufacturer sunflower kernels nutrition supplier. https://www.et-chem.com/sunflower-protein-manufacturer-sunflower-kernels-nutrition-supplier/ (2021).

[45] Tsaliki E, Pegiadou S, Doxastakis G (2004). Evaluation of the emulsifying properties of cottonseed protein isolates. Food Hydrocoll..

[46] Swiatkiewicz S, Arczewska-Wlosek A, Józefiak D (2016). The use of cottonseed meal as a protein source for poultry: an updated review. Worlds Poult. Sci. J..

[47] He Z, Zhang H, Olk DC (2015). Chemical composition of defatted cottonseed and soy meal products. PLoS ONE.

[48] Zhang B, Cui Y, Yin G, Li X, Zhou X (2009). Alkaline extraction method of cottonseed protein isolate. Mod. Appl. Sci..

[49] FAO. Definition and classification commodities, 4. Pulses and derived products. http://www.fao.org/es/faodef/fdef04e.htm (1984).

[50] Bessada SMF, Barreira JCM, Oliveira MBPP (2019). Pulses and food security: dietary protein, digestibility, bioactive and functional properties. Trends Food Sci. Technol..

[51] FAO. About the international year of pulses. http://www.fao.org/pulses-2016/about/en/ (2016).

[52] Boye J, Zare F, Pletch A (2010). Pulse proteins: processing, characterization, functional properties and applications in food and feed. Food Res. Int..

[53] Roquette. NUTRALYS® plant protein by Roquette. https://www.roquette.com/plant-protein (2021).

[54] Burcon. Peazazz. https://burcon.ca/products/peazazz/ (2021).

[55] Axiom Foods. Veg-o-tein neutral. http://axiomfoods.com/veg-o-tein-pea-protein-powder-neutral/ (2021).

[56] AMCO Proteins. Plant Proteins. https://www.amcoproteins.com/plant-proteins (2021).

[57] Ingredion. VITESSENCE® plant protein isolates. https://www.ingredion.com/na/en-us/ingredients/ingredient-product-families/vitessence-plant-protein-isolates.html (2021).

[58] Cargill. Pea protein - North America. https://www.cargill.com/food-bev/na/pea-protein (2021).

[59] Farbest Brands. Pea protein. https://farbest.com/ingredients/proteins/plant-proteins/pea-protein/ (2021).

[60] Nutrition—a plant based milk company. https://www.ripplefoods.com/protein-shake/ (2021).

[61] siggi’s. Icelandic yogurt—Vanilla plant based 24oz. https://siggis.com/categories/plant-based (2021).

[62] Montoya CA, Lallès JP, Beebe S, Leterme P (2010). Phaseolin diversity as a possible strategy to improve the nutritional value of common beans (Phaseolus vulgaris). Food Res. Int..

[63] Sathe SK (2002). Dry bean protein functionality. Crit. Rev. Biotechnol..

[64] ETprotein. Mung bean protein 80% innovated by ETprotein, China supplier. https://www.etprotein.com/mung-bean-protein-manufacturer-china-supplier/ (2021).

[65] Atura. Fava bean protein. https://aturaproteins.com/products/fava-bean-protein/ (2021).

[66] Green Boy. Mung bean powder. https://www.greenboyproducts.com/products/mung-bean/ (2021).

[67] Green Boy. Fava bean protein powder. https://www.greenboyproducts.com/products/fava-bean-protein-powder (2021).

[68] Nancy’s. Oatmilk non-dairy yogurt. https://nancysyogurt.com/products/oatmilk-non-dairy-yogurt/ (2021).

[69] Ghatak SK, Sen K (2013). Peanut proteins: applications, ailments and possible remediation. J. Ind. Eng. Chem..

[70] Wu H, Wang Q, Ma T, Ren J (2009). Comparative studies on the functional properties of various protein concentrate preparations of peanut protein. Food Res. Int..

[71] Liu Y, Zhao G, Zhao M, Ren J, Yang B (2012). Improvement of functional properties of peanut protein isolate by conjugation with dextran through Maillard reaction. Food Chem..

[72] He XH (2014). Effects of high pressure on the physicochemical and functional properties of peanut protein isolates. Food Hydrocoll..

[73] ETChem. Peanut protein powder peanut protein content. https://www.et-chem.com/peanut-protein-powder-peanut-protein-content-manufacturer/ (2021).

[74] Peanut Butter & Co. Peanut powder—Original. https://ilovepeanutbutter.com/products/peanut-powder-original (2021).

[75] InnovoPro. First in the world launching of chickpea protein in vegan protein powder. https://innovopro.com/first-in-the-world-launching-of-chickpea-protein-in-vegan-protein-powder/ (2021).

[76] Atura. Chickpea protein. https://aturaproteins.com/products/chickpea-protein/ (2021).

[77] Orgain. Organic plant protein | Vegan plant protein powder. https://orgain.com/collections/protein-powder/products/organic-simple-plant-protein-powder (2021).

[78] Manitoba Harvest. Hemp yeah! Plant protein blend unsweetened. https://manitobaharvest.com/products/hemp-yeah-plant-protein-blend-unsweetened (2021).

[79] Long, J., Ning, F. & Ning, C. CN105053506 (A). Preparation method of tea seed cake dreg isolated protein. https://worldwide.espacenet.com/publicationDetails/biblio?DB=EPODOC&adjacent=true&locale=en_EP&FT=D&date=20151118&CC=CN&NR=105053506A&KC=A (2015).

[80] Zhang, Z. & Zhang, K. CN104543328 (A). Preparation method and application for fermented gingko cell protein concentrated solution. https://worldwide.espacenet.com/publicationDetails/biblio?DB=EPODOC&adjacent=true&locale=en_EP&FT=D&date=20150429&CC=CN&NR=104543328A&KC=A (2015).

[81] Hoang, K. US10335446 (B2); US2019000910 (A1). Methods of treating diseases using grape proteins. https://worldwide.espacenet.com/publicationDetails/biblio?DB=EPODOC&adjacent=true&locale=en_EP&FT=D&date=20190103&CC=US&NR=2019000910A1&KC=A1 (2019).

[82] Wang, M., Yang, J., Wang, Y. & Zhu, S. CN110250276 (A). Fructus cannabis protein beverage and preparation method thereof. https://worldwide.espacenet.com/publicationDetails/biblio?DB=EPODOC&adjacent=true&locale=en_EP&FT=D&date=20190920&CC=CN&NR=110250276A&KC=A (2019).

[83] Samaranayaka, A. G. P., Wanasundara, U. N., Ray, M. & Green, R. C. WO2019213757 (A1). Hemp protein and use for microencapsulation. https://worldwide.espacenet.com/publicationDetails/biblio?DB=EPODOC&adjacent=true&locale=en_EP&FT=D&date=20191114&CC=WO&NR=2019213757A1&KC=A1 (2019).

[84] Wright, J. & Sprague, D. US2015079235 (A1). Hemp-based infant formula and Methods of Making Same. (2015). Available at: https://worldwide.espacenet.com/publicationDetails/biblio?DB=EPODOC&adjacent=true&locale=en_EP&FT=D&date=20150319&CC=US&NR=2015079235A1&KC=A1. (2015).

[85] Chen, Y. & Chen, Y. CN108522780 (A). Preparation method and application of industrial hempseed protein powder. https://worldwide.espacenet.com/publicationDetails/biblio?DB=EPODOC&adjacent=true&locale=en_EP&FT=D&date=20180914&CC=CN&NR=108522780A&KC=A (2018).

[86] Shi, J. et al. CN110150391 (A). Preparation method of sports type hemp kernel albumin beverage. https://worldwide.espacenet.com/publicationDetails/biblio?DB=EPODOC&adjacent=true&locale=en_EP&FT=D&date=20190823&CC=CN&NR=110150391A&KC=A (2019).

[87] Gosnell, B. & Schweizer, M. BR112015001964 (A2). Production of soluble protein products from hemp (‘H701’). https://worldwide.espacenet.com/publicationDetails/biblio?DB=EPODOC&adjacent=true&locale=en_EP&FT=D&date=20191217&CC=BR&NR=112015001964A2&KC=A2 (2019).

[88] Xiao, D. CN110856518 (A). Technological method for comprehensive treatment of Moringa oleifera seeds, product and application thereof. https://worldwide.espacenet.com/publicationDetails/biblio?DB=EPODOC&adjacent=true&locale=en_EP&FT=D&date=20200303&CC=CN&NR=110856518A&KC=A (2020).

[89] Chen, H. CN110463818 (A). Extraction method of protein in chili pepper seed meal and application. https://worldwide.espacenet.com/publicationDetails/biblio?DB=EPODOC&adjacent=true&locale=en_EP&FT=D&date=20191119&CC=CN&NR=110463818A&KC=A (2019).

[90] Wang, N. & Zhou, Q. CN110432333 (A). Preparation method of physalis alkekengi seed polysaccharide protein brewing product for reducing blood sugar. https://worldwide.espacenet.com/publicationDetails/biblio?DB=EPODOC&adjacent=true&locale=en_EP&FT=D&date=20191112&CC=CN&NR=110432333A&KC=A (2019).

[91] Bessonova, L. P., Antipova, L. V., Cherkasova, A. V. & Shapovalova, M. M. RU2590736 (C1). Method for production of food additive. https://worldwide.espacenet.com/publicationDetails/biblio?DB=EPODOC&adjacent=true&locale=en_EP&FT=D&date=20160710&CC=RU&NR=2590736C1&KC=C1 (2016).

[92] Hudson, C. J. & Hudson, S. P. BRPI0110757 (B1). Tryptophan source from plants and uses therefor. https://worldwide.espacenet.com/publicationDetails/biblio?DB=EPODOC&adjacent=true&locale=en_EP&FT=D&date=20171128&CC=BR&NR=PI0110757B1&KC=B1 (2017).

[93] Helling, R. K., Patterson, T. G. & Campbell, S. J. AR097442 (A2). Aqueous processing of oilseed press cake. https://worldwide.espacenet.com/publicationDetails/biblio?DB=EPODOC&adjacent=true&locale=en_EP&FT=D&date=20160316&CC=AR&NR=097442A2&KC=A2 (2016).

[94] Hiron, S. BRPI0308380 (B1). Canola protein isolate functionality III. https://worldwide.espacenet.com/publicationDetails/biblio?DB=EPODOC&adjacent=true&locale=en_EP&FT=D&date=20181204&CC=BR&NR=PI0308380B1&KC=B1 (2018).

[95] Green, B. E. et al. BR112012008321 (A2). Canola protein product from supernatant. https://worldwide.espacenet.com/publicationDetails/biblio?DB=EPODOC&adjacent=true&locale=en_EP&FT=D&date=20170516&CC=BR&NR=112012008321A2&KC=A2 (2017).

[96] Shi, J., Smolders, G. J. F., Willemsen, J. H. M., Vermunt, J. H. A. J. & Hylkema, N. N. US2020154732 (A1). Gluten free native rapeseed protein isolate. https://worldwide.espacenet.com/publicationDetails/biblio?DB=EPODOC&adjacent=true&locale=en_EP&FT=D&date=20200521&CC=US&NR=2020154732A1&KC=A1 (2020).

[97] Feng, L. CN107873944 (A). Industrial production of rapeseed active peptide and preparation method. https://worldwide.espacenet.com/publicationDetails/biblio?DB=EPODOC&adjacent=true&locale=en_EP&FT=D&date=20180406&CC=CN&NR=107873944A&KC=A (2018).

[98] Vlasie, M. D. WO2019234137 (A1). Modified rapeseed protein isolate. https://worldwide.espacenet.com/publicationDetails/biblio?DB=EPODOC&adjacent=true&locale=en_EP&FT=D&date=20191212&CC=WO&NR=2019234137A1&KC=A1 (2019).

[99] Green, B. E., Segall, K. I., Schweizer, M. & Willardsen, R. BRPI0913429 (A2). Novel canola protein isolate. https://worldwide.espacenet.com/publicationDetails/biblio?DB=EPODOC&adjacent=true&locale=en_EP&FT=D&date=20151124&CC=BR&NR=PI0913429A2&KC=A2 (2015).

[100] Segall, K. I., Green, B. E. & Schweizer, M. BRPI0917301 (A2). Preparation of canola protein isolate from canola oil seeds (‘Blendertein’). https://worldwide.espacenet.com/publicationDetails/biblio?DB=EPODOC&adjacent=true&locale=en_EP&FT=D&date=20150728&CC=BR&NR=PI0917301A2&KC=A2 (2015).

[101] Segall, K. I., Green, B. E. & Schweizer, M. BRPI0917304 (A2). Preparation of canola protein isolate without heat treatment (‘C200Ca’). https://worldwide.espacenet.com/publicationDetails/biblio?DB=EPODOC&adjacent=true&locale=en_EP&FT=D&date=20150728&CC=BR&NR=PI0917304A2&KC=A2 (2015).

[102] Segall, K. I., Green, B. E. & Schweizer, M. PL2323499 (T3). Production of canola protein isolate without heat treatment. https://worldwide.espacenet.com/publicationDetails/biblio?DB=EPODOC&adjacent=true&locale=en_EP&FT=D&date=20191231&CC=PL&NR=2323499T3&KC=T3 (2019).

[103] Green, B. E., Segall, K. I., Schweizer, M. & Medina, S. BRPI1012171 (A2); BRPI1012171 (B1). Production of canola protein product without heat treatment (‘C200CaC’). https://worldwide.espacenet.com/publicationDetails/biblio?DB=EPODOC&adjacent=true&locale=en_EP&FT=D&date=20161025&CC=BR&NR=PI1012171A2&KC=A2 (2016).

[104] Green, B. E., Logie, J., Segall, K. I. & Schweizer, M. BRPI0915489 (A2); BRPI0915489 (B1). Soluble canola protein isolate production (‘Nutratein’). https://worldwide.espacenet.com/publicationDetails/biblio?DB=EPODOC&adjacent=true&locale=en_EP&FT=D&date=20190108&CC=BR&NR=PI0915489A2&KC=A2 (2019).

[105] Segall, K. I., Green, B. E. & Schweizer, M. BRPI0917295 (A2). Soluble canola protein isolate production from PMM (‘C307’). https://worldwide.espacenet.com/publicationDetails/biblio?DB=EPODOC&adjacent=true&locale=en_EP&FT=D&date=20150728&CC=BR&NR=PI0917295A2&KC=A2 (2015).

[106] Shi, J. & Smolders, G. J. F. EP3481220 (A1). Sweet rapeseed protein isolate and process for obtaining it. https://worldwide.espacenet.com/publicationDetails/biblio?DB=EPODOC&adjacent=true&locale=en_EP&FT=D&date=20190515&CC=EP&NR=3481220A1&KC=A1 (2019).

[107] Van den Berg, M. & Shi, J. WO2019110556 (A1). Sweet rapeseed protein isolate. https://worldwide.espacenet.com/publicationDetails/biblio?DB=EPODOC&adjacent=true&locale=en_EP&FT=D&date=20190613&CC=WO&NR=2019110556A1&KC=A1 (2019).

[108] Tang, Q. N. HUE033952 (T2). Oilseed protein concentrates and processes for the production thereof. https://worldwide.espacenet.com/publicationDetails/biblio?DB=EPODOC&adjacent=true&locale=en_EP&FT=D&date=20180129&CC=HU&NR=E033952T2&KC=T2 (2018).

[109] Tang, Q. N. PL2498620 (T3). Protein concentrates and isolates, and processes for the production thereof. https://worldwide.espacenet.com/publicationDetails/biblio?DB=EPODOC&adjacent=true&locale=en_EP&FT=D&date=20171031&CC=PL&NR=2498620T3&KC=T3 (2017).

[110] Zhang, L. et al. CN104543326 (A). Samara protein powder and method for preparing same. https://worldwide.espacenet.com/publicationDetails/biblio?DB=EPODOC&adjacent=true&locale=en_EP&FT=D&date=20150429&CC=CN&NR=104543326A&KC=A (2015).

[111] Li, H. et al. CN109430515 (A). Method for preparing fermented sesame seed protein by using probiotics and application of fermented sesame seed protein. https://worldwide.espacenet.com/publicationDetails/biblio?DB=EPODOC&adjacent=true&locale=en_EP&FT=D&date=20190308&CC=CN&NR=109430515A&KC=A (2019).

[112] Chen, Y. et al. CN110810687 (A). Method for preparing oligopeptide refreshing beverage by using endogenous endopeptidase and exopeptidase to hydrolyze sesame protein. https://worldwide.espacenet.com/publicationDetails/biblio?DB=EPODOC&adjacent=true&locale=en_EP&FT=D&date=20200221&CC=CN&NR=110810687A&KC=A (2020).

[113] Wang, C. CN105192244 (A). Method for preparing snakegourd fruit seed polypeptides. https://worldwide.espacenet.com/publicationDetails/biblio?DB=EPODOC&adjacent=true&locale=en_EP&FT=D&date=20151230&CC=CN&NR=105192244A&KC=A (2015).

[114] Pscheidl, M. & Hammerl, P. HUE029430 (T2). Method and system for extraction of vegetable protein, in particular as protein-rich foodstuff or animal feed, and protein-rich human and animal food. https://worldwide.espacenet.com/publicationDetails/biblio?DB=EPODOC&adjacent=true&locale=en_EP&FT=D&date=20170328&CC=HU&NR=E029430T2&KC=T2 (2017).

[115] Zheng, J. et al. CN109354574 (A). Method for preparing sunflower seed chlorogenic acid and protein powder. https://worldwide.espacenet.com/publicationDetails/biblio?DB=EPODOC&adjacent=true&locale=en_EP&FT=D&date=20190219&CC=CN&NR=109354574A&KC=A (2019).

[116] Manchuliantsau, A. & Tkacheva, A. CN110678083 (A). High-protein oilcake-based nutritional composition. https://worldwide.espacenet.com/publicationDetails/biblio?DB=EPODOC&adjacent=true&locale=en_EP&FT=D&date=20200110&CC=CN&NR=110678083A&KC=A (2020).

[117] German, A. I. RU2538147 (C1). Method for processing of sunflower or rape extraction cake (versions). https://worldwide.espacenet.com/publicationDetails/biblio?DB=EPODOC&adjacent=true&locale=en_EP&FT=D&date=20150110&CC=RU&NR=2538147C1&KC=C1 (2015).

[118] Zhu, S. et al. CN109266432 (A). Method for synchronously extracting silybum marianum oil and hydrolyzed protein. https://worldwide.espacenet.com/publicationDetails/biblio?DB=EPODOC&adjacent=true&locale=en_EP&FT=D&date=20190125&CC=CN&NR=109266432A&KC=A (2019).

[119] Ning, D. et al. CN209711374 (U). Walnut protein powder preparation system. https://worldwide.espacenet.com/publicationDetails/biblio?DB=EPODOC&adjacent=true&locale=en_EP&FT=D&date=20191203&CC=CN&NR=209711374U&KC=U (2019).

[120] Kizer, L., Renninger, N. & Stiles, A. US2019000112 (A1). Product analogs or components of such analogs and processes for making same. https://worldwide.espacenet.com/publicationDetails/biblio?DB=EPODOC&adjacent=true&locale=en_EP&FT=D&date=20190103&CC=US&NR=2019000112A1&KC=A1 (2019).

[121] Ayoub, A., Ghotra, B. & Richardson, J. EP3573471 (A1). Novel thickening compositions based on starch. https://worldwide.espacenet.com/publicationDetails/biblio?DB=EPODOC&adjacent=true&locale=en_EP&FT=D&date=20191204&CC=EP&NR=3573471A1&KC=A1 (2019).

[122] Jung, L. E., Nam, L. Il & Jun, J. H. US2019133150 (A1). Protein-containing powder and method of producing thereof. https://worldwide.espacenet.com/publicationDetails/biblio?DB=EPODOC&adjacent=true&locale=en_EP&FT=D&date=20190509&CC=US&NR=2019133150A1&KC=A1 (2019).

[123] Vadlamani, K. R. US2019239535 (A1). Black-eyed pea protein isolates, products, and methods. https://worldwide.espacenet.com/publicationDetails/biblio?DB=EPODOC&adjacent=true&locale=en_EP&FT=D&date=20190808&CC=US&NR=2019239535A1&KC=A1 (2019).

[124] Mcfarlane, P., Mckeegan, B., Lingham, C. & Lingham, R. WO2020051622 (A1). System and method for extracting a protein food product. https://worldwide.espacenet.com/publicationDetails/biblio?DB=EPODOC&adjacent=true&locale=en_EP&FT=D&date=20200319&CC=WO&NR=2020051622A1&KC=A1 (2020).

[125] Bar-El Dadon, S., Reifen, R. & Schuring, M. JP2019520082 (A). Chickpea protein concentrate. https://worldwide.espacenet.com/publicationDetails/biblio?DB=EPODOC&adjacent=true&locale=en_EP&FT=D&date=20190718&CC=JP&NR=2019520082A&KC=A (2019).

[126] Lu, X., Wang, B., Yao, L., Wang, L. N. & Wu, T. CN104774271 (A); CN104774271 (B). Preparation method of white hyacinth bean extract powder. https://worldwide.espacenet.com/publicationDetails/biblio?DB=EPODOC&adjacent=true&locale=en_EP&FT=D&date=20150715&CC=CN&NR=104774271A&KC=A (2015).

[127] Sub, J. Y. KR102092404 (B1); KR20200013276 (A). Vegetable milk composition manufacturing method thereof and ice creams containing it. https://worldwide.espacenet.com/publicationDetails/biblio?DB=EPODOC&adjacent=true&locale=en_EP&FT=D&date=20200207&CC=KR&NR=20200013276A&KC=A (2020).

[128] Bialek, J. M., Van Der Hijden, H. T. W. M., Khalloufi, S., Nieman, G. & Vreeker, R. EP3209141 (A1); EP3209141 (B1). Lentil-derived foaming agent and foamable compositions containing such foaming agent. https://worldwide.espacenet.com/publicationDetails/biblio?DB=EPODOC&adjacent=true&locale=en_EP&FT=D&date=20170830&CC=EP&NR=3209141A1&KC=A1 (2017).

[129] Medina, S. WO2020061698 (A1). pH adjusted pulse protein product. https://worldwide.espacenet.com/publicationDetails/biblio?DB=EPODOC&adjacent=true&locale=en_EP&FT=D&date=20200402&CC=WO&NR=2020061698A1&KC=A1 (2020).

[130] Segall, K. I. & Schweizer, M. BR112015007140 (A2). Production of pulse protein product using calcium chloride extraction (‘yp702’). https://worldwide.espacenet.com/publicationDetails/biblio?DB=EPODOC&adjacent=true&locale=en_EP&FT=D&date=20191217&CC=BR&NR=112015007140A2&KC=A2 (2019).

[131] Green, B. E., Schweizer, M. & Sampson, R. KR20190087654 (A). Production of pulse protein product. https://worldwide.espacenet.com/publicationDetails/biblio?DB=EPODOC&adjacent=true&locale=en_EP&FT=D&date=20190724&CC=KR&NR=20190087654A&KC=A (2019).

[132] Schweizer, M., Medina, S. & Segall, K. I. EP3586644 (A1). Production of pulse protein products with reduced astringency. https://worldwide.espacenet.com/publicationDetails/biblio?DB=EPODOC&adjacent=true&locale=en_EP&FT=D&date=20200101&CC=EP&NR=3586644A1&KC=A1 (2020).

[133] Segall, K. I. & Schweizer, M. JP2018110600 (A). Production of soluble protein solutions from pulses. https://worldwide.espacenet.com/publicationDetails/biblio?DB=EPODOC&adjacent=true&locale=en_EP&FT=D&date=20180719&CC=JP&NR=2018110600A&KC=A (2018).

[134] Eisner, P. et al. CA2953644 (A1). Emulsion with lupine protein. https://worldwide.espacenet.com/publicationDetails/biblio?DB=EPODOC&adjacent=true&locale=en_EP&FT=D&date=20160107&CC=CA&NR=2953644A1&KC=A1 (2016).

[135] Kuznetsova, L. M., Zabodalova, L. A., Domoroshchenkova, M. L. V. & Dem Janenko, T. J. F. RU2555528 (C1). Vegetal raw material based product manufacture method. https://worldwide.espacenet.com/publicationDetails/biblio?DB=EPODOC&adjacent=true&locale=en_EP&FT=D&date=20150710&CC=RU&NR=2555528C1&KC=C1 (2015).

[136] Bansal-Mutalik, R. et al. US2019191735 (A1). Functonal mung bean-deriver compositions. https://worldwide.espacenet.com/publicationDetails/biblio?DB=EPODOC&adjacent=true&locale=en_EP&FT=D&date=20190627&CC=US&NR=2019191735A1&KC=A1 (2019).

[137] Motoyama, T. JP6521257 (B2); JPWO2015105138 (A1). Mung bean protein composition. https://worldwide.espacenet.com/publicationDetails/biblio?DB=EPODOC&adjacent=true&locale=en_EP&FT=D&date=20170323&CC=JP&NR=WO2015105138A1&KC=A1 (2017).

[138] Motoyama, T. & Ashida, S. JP6332266 (B2); JPWO2014156549 (A1). Mung bean protein gel composition and cheese-like food. https://worldwide.espacenet.com/publicationDetails/biblio?DB=EPODOC&adjacent=true&locale=en_EP&FT=D&date=20170216&CC=JP&NR=WO2014156549A1&KC=A1 (2017).

[139] Zhang, P. & Su, P. CN106261782 (A). Processing method for manufacturing different functional food through mungbean biofermentation separation and whole utilization. https://worldwide.espacenet.com/publicationDetails/biblio?DB=EPODOC&adjacent=true&locale=en_EP&FT=D&date=20170104&CC=CN&NR=106261782A&KC=A (2017).

[140] Amazan, M. CA2982280 (A1). Plant-based legume milk alternative and other consumable products using same. https://worldwide.espacenet.com/publicationDetails/biblio?DB=EPODOC&adjacent=true&locale=en_EP&FT=D&date=20190408&CC=CA&NR=2982280A1&KC=A1 (2019).

[141] Kizer, L., Renninger, N. & Schelle, M. MX2018012893 (A). Dairy product analogs and processes for making same. https://worldwide.espacenet.com/publicationDetails/biblio?DB=EPODOC&adjacent=true&locale=en_EP&FT=D&date=20190610&CC=MX&NR=2018012893A&KC=A (2019).

[142] Hossen, M., Pinkston, J. D., Tenea, A. R. & Cherian, G. CA3067593 (A1). Deflavored pea composition. https://worldwide.espacenet.com/publicationDetails/biblio?DB=EPODOC&adjacent=true&locale=en_EP&FT=D&date=20190103&CC=CA&NR=3067593A1&KC=A1 (2019).

[143] Ito, G. WO2020007940 (A1). Food composition comprising plant proteins and a potassium metaphosphate. https://worldwide.espacenet.com/publicationDetails/biblio?DB=EPODOC&adjacent=true&locale=en_EP&FT=D&date=20200109&CC=WO&NR=2020007940A1&KC=A1 (2020).

[144] Manuel, B. et al. FR3047151 (A1). Formulations nutritionnelles comprenant un isolat de proteines de pois. https://worldwide.espacenet.com/publicationDetails/biblio?DB=EPODOC&adjacent=true&locale=en_EP&FT=D&date=20170804&CC=FR&NR=3047151A1&KC=A1 (2017).

[145] Lecocq, A., Senecot, L. & Barata, M. BR112019022289 (A2). Improved pea albumins, method for obtaining same and applications thereof. https://worldwide.espacenet.com/publicationDetails/biblio?DB=EPODOC&adjacent=true&locale=en_EP&FT=D&date=20200519&CC=BR&NR=112019022289A2&KC=A2 (2020).

[146] Lin, F. CN107668314 (A). Industrially produced pea bioactive peptides and preparation method thereof. https://worldwide.espacenet.com/publicationDetails/biblio?DB=EPODOC&adjacent=true&locale=en_EP&FT=D&date=20180209&CC=CN&NR=107668314A&KC=A (2018).

[147] Bourgeois, A., Gramain, A. & Descamps, M. US2016316785 (A1). Method for extracting pea proteins. https://worldwide.espacenet.com/publicationDetails/biblio?DB=EPODOC&adjacent=true&locale=en_EP&FT=D&date=20161103&CC=US&NR=2016316785A1&KC=A1 (2016).

[148] Ito, G. FR3070831 (A1). Method for preparing a composition based on legume proteins. https://worldwide.espacenet.com/publicationDetails/biblio?DB=EPODOC&adjacent=true&locale=en_EP&FT=D&date=20190315&CC=FR&NR=3070831A1&KC=A1 (2019).

[149] Zhao, J., Lin, J., Cao, J., Zhao, J. & Zhao, Y. CN109907156 (A). Method for producing brewing type pea protein isolate finished product. https://worldwide.espacenet.com/publicationDetails/biblio?DB=EPODOC&adjacent=true&locale=en_EP&FT=D&date=20190621&CC=CN&NR=109907156A&KC=A (2019).

[150] Guillemant, M., Delebarre, M., Barata, M., Moretti, E. & Muller, E. MX2018010758 (A). Nutritional formulations such as a yoghurt, cream, cream dessert or frozen dessert, comprising a pea protein isolate, and the use of the formulation as a source of protein. https://worldwide.espacenet.com/publicationDetails/biblio?DB=EPODOC&adjacent=true&locale=en_EP&FT=D&date=20190520&CC=MX&NR=2018010758A&KC=A (2019).

[151] Lecocq, A. & Ibert, M. CO2020004049 (A2). Pea protein composition having improved nutritional quality. https://worldwide.espacenet.com/publicationDetails/biblio?DB=EPODOC&adjacent=true&locale=en_EP&FT=D&date=20200529&CC=CO&NR=2020004049A2&KC=A2 (2020).

[152] Han, S., Ismail, P., Ties, P. & Mitacek, R. CA3079976 (A1). Pea protein hidrolysate. https://worldwide.espacenet.com/publicationDetails/biblio?DB=EPODOC&adjacent=true&locale=en_EP&FT=D&date=20190509&CC=CA&NR=3079976A1&KC=A1 (2019).

[153] Chandak, K. N. & Lorenzen, T. J. US2019053517 (A1). Pea protein product. https://worldwide.espacenet.com/publicationDetails/biblio?DB=EPODOC&adjacent=true&locale=en_EP&FT=D&date=20190221&CC=US&NR=2019053517A1&KC=A1 (2019).

[154] Liu, L. et al. CN109566747 (A). Pea protein vegan plant-based yoghurt and preparation method thereof. https://worldwide.espacenet.com/publicationDetails/biblio?DB=EPODOC&adjacent=true&locale=en_EP&FT=D&date=20190405&CC=CN&NR=109566747A&KC=A (2019).

[155] Gosnell, B., Segall, K., Schweizer, M., Willardsen, R. & Medina, S. BR112019018323 (A2). Preparation of acid soluble pulse protein hydrolyzates with little or no astringency and pulse protein hydrolyzates of improved amino acid score. https://worldwide.espacenet.com/publicationDetails/biblio?DB=EPODOC&adjacent=true&locale=en_EP&FT=D&date=20200331&CC=BR&NR=112019018323A2&KC=A2 (2020).

[156] Barata, M., Duflot, P., Dhalleine, C. & Verrin, J.-M. ES2676925 (T3). Procedimiento de fraccionamiento de las fracciones solubles de guisante. https://worldwide.espacenet.com/publicationDetails/biblio?DB=EPODOC&adjacent=true&locale=en_EP&FT=D&date=20180726&CC=ES&NR=2676925T3&KC=T3 (2018).

[157] Zhao, J., Lin, J., Cao, J., Zhao, J. & Zhao, Y. CN109907155 (A). Production process of brewing type pea protein isolate powder. https://worldwide.espacenet.com/publicationDetails/biblio?DB=EPODOC&adjacent=true&locale=en_EP&FT=D&date=20190621&CC=CN&NR=109907155A&KC=A (2019).

[158] Lihme, A. O. F., Hansen, M. B. & Pontoppidan, M. EA030803 (B1); EA201691510 (A1). Separation processes for pea protein. https://worldwide.espacenet.com/publicationDetails/biblio?DB=EPODOC&adjacent=true&locale=en_EP&FT=D&date=20161230&CC=EA&NR=201691510A1&KC=A1 (2016).

[159] Ventureira, J. L. WO2020109741 (A1). Soluble legume protein. https://worldwide.espacenet.com/publicationDetails/biblio?DB=EPODOC&adjacent=true&locale=en_EP&FT=D&date=20200604&CC=WO&NR=2020109741A1&KC=A1 (2020).

[160] King, A. E., Novak, D. R., Phillips, J. T., Atchison, N. A. & Chandak, K. N. US2020100524 (A1). Soluble pea protein products. https://worldwide.espacenet.com/publicationDetails/biblio?DB=EPODOC&adjacent=true&locale=en_EP&FT=D&date=20200402&CC=US&NR=2020100524A1&KC=A1 (2020).

[161] Foster, S. A., Foster, T. G. & Crank, D. L. CA3008464 (A1). Yellow pea protein compositions with high digestibilities and amino acid scores. https://worldwide.espacenet.com/publicationDetails/biblio?DB=EPODOC&adjacent=true&locale=en_EP&FT=D&date=20190715&CC=CA&NR=3008464A1&KC=A1 (2019).

[162] Wang, F., Zhang, Y., Xu, L. & Ma, H. CN111066944 (A). Method for improving vicilin functional properties. https://worldwide.espacenet.com/publicationDetails/biblio?DB=EPODOC&adjacent=true&locale=en_EP&FT=D&date=20200428&CC=CN&NR=111066944A&KC=A (2020).

[163] Theil, E. C. US2017087209 (A1). Methods for isolation, use and analysis of ferritin. https://worldwide.espacenet.com/publicationDetails/biblio?DB=EPODOC&adjacent=true&locale=en_EP&FT=D&date=20170330&CC=US&NR=2017087209A1&KC=A1 (2017).

[164] Lu, X. et al. CN106260496 (A). Method for improving solubility of peanut protein. https://worldwide.espacenet.com/publicationDetails/biblio?DB=EPODOC&adjacent=true&locale=en_EP&FT=D&date=20170104&CC=CN&NR=106260496A&KC=A (2017).

[165] Liu, Y. & Li, K. CN106367196 (A); CN106367196 (B). Method for obtaining peanut oil with soft and refined fragrance and high-quality protein powder simultaneously. https://worldwide.espacenet.com/publicationDetails/biblio?DB=EPODOC&adjacent=true&locale=en_EP&FT=D&date=20170201&CC=CN&NR=106367196A&KC=A (2017).

[166] Peng, X. CN107927319 (A). Method for preparing peanut polypeptide solution by utilizing peanut intrinsic protease to hydrolyze peanut protein. https://worldwide.espacenet.com/publicationDetails/biblio?DB=EPODOC&adjacent=true&locale=en_EP&FT=D&date=20180420&CC=CN&NR=107927319A&KC=A (2018).

[167] Wang, Q. et al. CN110140801 (A). Method for simultaneously preparing high-oleic-acid peanut oil and peanut albumen powder. https://worldwide.espacenet.com/publicationDetails/biblio?DB=EPODOC&adjacent=true&locale=en_EP&FT=D&date=20190820&CC=CN&NR=110140801A&KC=A (2019).

[168] Wang, Q. et al. US2020170279 (A1). Pickering emulsion prepared using peanut protein isolate and preparation method thereof. https://worldwide.espacenet.com/publicationDetails/biblio?DB=EPODOC&adjacent=true&locale=en_EP&FT=D&date=20200604&CC=US&NR=2020170279A1&KC=A1 (2020).

[169] Lan, X. CN106333057 (A). Preparation method for peanut meal protein isolate. https://worldwide.espacenet.com/publicationDetails/biblio?DB=EPODOC&adjacent=true&locale=en_EP&FT=D&date=20170118&CC=CN&NR=106333057A&KC=A (2017).

[170] Lorand, J.-P. & Basse, B. FR3072002 (A1); FR3072002 (B1). Procede de preparation d’un gel alimentaire de proteines d’arachide, gel obtenu et son utilisation. https://worldwide.espacenet.com/publicationDetails/biblio?DB=EPODOC&adjacent=true&locale=en_EP&FT=D&date=20190412&CC=FR&NR=3072002A1&KC=A1 (2019).

[171] Fári, M. G. & Domokos-Szabolcsy, É. WO2019150144 (A1). Method for producing plant protein coagulum. https://worldwide.espacenet.com/publicationDetails/biblio?DB=EPODOC&adjacent=true&locale=en_EP&FT=D&date=20190808&CC=WO&NR=2019150144A1&KC=A1 (2019).

[172] Brown, P., Casino, M., Voccola, L. S. & Varadan, R. CN110742128 (A). Methods and compositions for consumables. https://worldwide.espacenet.com/publicationDetails/biblio?DB=EPODOC&adjacent=true&locale=en_EP&FT=D&date=20200204&CC=CN&NR=110742128A&KC=A (2020).

[173] Dierbach, L. A., High, R. M., Kimmel, J. L., Laudano, R. & Ortiz, I. A. EP3429366 (A1). Stable protein products and methods for making the same. https://worldwide.espacenet.com/publicationDetails/biblio?DB=EPODOC&adjacent=true&locale=en_EP&FT=D&date=20190123&CC=EP&NR=3429366A1&KC=A1 (2019).

[174] Zemach, A. M. US2019225645 (A1). Method for preparing a complete protein and uses thereof. https://worldwide.espacenet.com/publicationDetails/biblio?DB=EPODOC&adjacent=true&locale=en_EP&FT=D&date=20190725&CC=US&NR=2019225645A1&KC=A1 (2019).

[175] Liu, X., Ding, Z., Wang, S., Lin, A. & Guo, Z. CN110637916 (A). Edible fermentation short chain polypeptide and preparation method thereof. https://worldwide.espacenet.com/publicationDetails/biblio?DB=EPODOC&adjacent=true&locale=en_EP&FT=D&date=20200103&CC=CN&NR=110637916A&KC=A (2020).

[176] Scotland, R. & Liu, X. JP2017521498 (A). Isolation of plant oligopeptides and uses thereof. https://worldwide.espacenet.com/publicationDetails/biblio?DB=EPODOC&adjacent=true&locale=en_EP&FT=D&date=20170803&CC=JP&NR=2017521498A&KC=A (2017).

[177] Zhang, H. K., Gray, J. A., Chavez, L. M. & Behr, W. K. EP3634146 (A1). Systems and methods using physical energy technology to produce non-dairy protein base and value-added utilization of the co-product. https://worldwide.espacenet.com/publicationDetails/biblio?DB=EPODOC&adjacent=true&locale=en_EP&FT=D&date=20200415&CC=EP&NR=3634146A1&KC=A1 (2020).

[178] Bunce, M. G., Saffon, M., Fu, J.-T. & Sher, A. US2019307143 (A1). Liquid coconut-based coffee creamer and method of making the same. https://worldwide.espacenet.com/publicationDetails/biblio?DB=EPODOC&adjacent=true&locale=en_EP&FT=D&date=20191010&CC=US&NR=2019307143A1&KC=A1 (2019).

[179] Liu, Z., Du, Y. & Wang, S. CN111227101 (A). Albumen product and preparation method thereof. https://worldwide.espacenet.com/publicationDetails/biblio?DB=EPODOC&adjacent=true&locale=en_EP&FT=D&date=20200605&CC=CN&NR=111227101A&KC=A (2020).

[180] Riihinen, K. FI128029 (B). Process for producing a plant protein ingredient. https://worldwide.espacenet.com/publicationDetails/biblio?DB=EPODOC&adjacent=true&locale=en_EP&FT=D&date=20190815&CC=FI&NR=128029B&KC=B (2019).

[181] Rogé, V. J. FR3085826 (A3). Composition végane sous forme de poudre. https://worldwide.espacenet.com/publicationDetails/biblio?DB=EPODOC&adjacent=true&locale=en_EP&FT=D&date=20200320&CC=FR&NR=3085826A3&KC=A3 (2020).

[182] Zhang, Y. & Potts, D. WO2019228957 (A1). Organic compounds. https://worldwide.espacenet.com/publicationDetails/biblio?DB=EPODOC&adjacent=true&locale=en_EP&FT=D&date=20191205&CC=WO&NR=2019228957A1&KC=A1 (2019).

[183] Keitzel, A. et al. BR112018070543 (A2). Techno-functional plant protein fraction from leguminous or oil seeds. https://worldwide.espacenet.com/publicationDetails/biblio?DB=EPODOC&adjacent=true&locale=en_EP&FT=D&date=20190212&CC=BR&NR=112018070543A2&KC=A2 (2019).

[184] Guerrier, J.-L., Erabit, N. & Guillemot, P. BR112019019992 (A2). Hydrolysed vegetable proteins suitable for use in baby food. https://worldwide.espacenet.com/publicationDetails/biblio?DB=EPODOC&adjacent=true&locale=en_EP&FT=D&date=20200428&CC=BR&NR=112019019992A2&KC=A2 (2020).

[185] Schmidt, L. et al. US2020060310 (A1). Myceliated vegetable protein and food compositions comprising same. https://worldwide.espacenet.com/publicationDetails/biblio?DB=EPODOC&adjacent=true&locale=en_EP&FT=D&date=20200227&CC=US&NR=2020060310A1&KC=A1 (2020).

[186] Lorand, J. P., Kandil, L. & D’huart, J. B. FR3019004 (A1); FR3019004 (B1). Foodstuff comprising proteins essentialy of vegetable origin and process for preparing same. https://worldwide.espacenet.com/publicationDetails/biblio?DB=EPODOC&adjacent=true&locale=en_EP&FT=D&date=20151002&CC=FR&NR=3019004A1&KC=A1 (2015).

[187] Chen, F. et al. CN107125430 (A). Method for simultaneously preparing oil body and non-hydrolyzed protein. https://worldwide.espacenet.com/publicationDetails/biblio?DB=EPODOC&adjacent=true&locale=en_EP&FT=D&date=20170905&CC=CN&NR=107125430A&KC=A (2017).

[188] Segall, K. I., Schweizer, M. & Green, B. E. RU2018129466 (A). Preparation of non-soy oilseed protein products (‘ ‘*810”). https://worldwide.espacenet.com/publicationDetails/biblio?DB=EPODOC&adjacent=true&locale=en_EP&FT=D&date=20200228&CC=RU&NR=2018129466A&KC=A (2020).

[189] Manchuliantsau, A. & Tkacheva, A. WO2020028446 (A1). Upcycling solid food wastes and by-products into food-grade nutritional products. https://worldwide.espacenet.com/publicationDetails/biblio?DB=EPODOC&adjacent=true&locale=en_EP&FT=D&date=20200206&CC=WO&NR=2020028446A1&KC=A1 (2020).

[190] Manchuliantsau, A. & Tkacheva, A. US10645950 (B2); US2020060308 (A1). Methods of manufacturing products from material comprising oilcake, compositions produced from materials comprising processed oilcake, and systems for processing oilcake. https://worldwide.espacenet.com/publicationDetails/biblio?DB=EPODOC&adjacent=true&locale=en_EP&FT=D&date=20200227&CC=US&NR=2020060308A1&KC=A1 (2020).

[191] Ido, H., Obara, T. & Yamaguchi, S. JP6059659 (B2); JPWO2013027813 (A1). Composition having improved foam stability and use thereof. https://worldwide.espacenet.com/publicationDetails/biblio?DB=EPODOC&adjacent=true&locale=en_EP&FT=D&date=20150319&CC=JP&NR=WO2013027813A1&KC=A1 (2015).

